# *Arabidopsis* COP1 SUPPRESSOR 2 Represses COP1 E3 Ubiquitin Ligase Activity through Their Coiled-Coil Domains Association

**DOI:** 10.1371/journal.pgen.1005747

**Published:** 2015-12-29

**Authors:** Dongqing Xu, Fang Lin, Yan Jiang, Junjie Ling, Chamari Hettiarachchi, Christian Tellgren-Roth, Magnus Holm, Ning Wei, Xing Wang Deng

**Affiliations:** 1 State Key Laboratory of Protein and Plant Gene Research, Peking-Tsinghua Center for Life Sciences, School of Advanced Agriculture Sciences and School of Life Sciences, Peking University, Beijing, China; 2 Department of Biological and Environmental Sciences, Gothenburg University, Gothenburg, Sweden; 3 Department of Chemistry, University of Colombo, Colombo, Sri Lanka; 4 Uppsala Genome Center, National Genomics Infrastructure, Science for Life Laboratory, Department of Immunology, Genetics and Pathology, Uppsala University, BMC, Uppsala, Sweden; 5 Department of Molecular, Cellular, and Developmental Biology, Yale University, New Haven, Connecticut, United States of America; University of California Riverside, UNITED STATES

## Abstract

CONSTITUTIVE PHOTOMORPHOGENIC 1 (COP1) functions as an E3 ubiquitin ligase and mediates a variety of developmental processes in *Arabidopsis* by targeting a number of key regulators for ubiquitination and degradation. Here, we identify a novel COP1 interacting protein, COP1 SUPPRESSOR 2 (CSU2). Loss of function mutations in *CSU2* suppress the constitutive photomorphogenic phenotype of *cop1-6* in darkness. CSU2 directly interacts with COP1 via their coiled-coil domains and is recruited by COP1 into nuclear speckles in living plant cells. Furthermore, CSU2 inhibits COP1 E3 ubiquitin ligase activity *in vitro*, and represses COP1 mediated turnover of HY5 in cell-free extracts. We propose that in *csu2 cop1-6* mutants, the lack of CSU2’s repression of COP1 allows the low level of COP1 to exhibit higher activity that is sufficient to prevent accumulation of HY5 in the dark, thus restoring the etiolated phenotype. In addition, CSU2 is required for primary root development under normal light growth condition.

## Author Summary

CONSTITUTIVE PHOTOMORPHOGENIC 1 (COP1) is a key regulator of light mediated developmental processes and it works as an E3 ubiquitin ligase controlling the abundance of multiple transcription factors. In the work presented here, we identified a novel repressor of COP1, the COP1 SUPPRESSOR 2 (CSU2), via a forward genetic screen. Mutations in *CSU2* completely suppress *cop1-6* constitutive photomorphogenic phenotype in darkness. CSU2 interacts and co-localizes with COP1 in nuclear speckles via the coiled-coil domain association. CSU2 negatively regulates COP1 E3 ubiquitin ligase activity, and repress COP1 mediated HY5 degradation in cell-free extracts.

## Introduction

Sunlight provides not only the major energy source, but also a main environmental signal that regulates multiple developmental processes in plants, such as seed germination, photomorphogenesis, flowering, phototropism and root growth [1]. In *Arabidopsis thaliana*, phytochromes (phyA-phyE) sense red and far-red light (600–750 nm) [2, 3]; cryptochromes (CRY1 and CRY2) and phototropins (PHOT1 and PHOT2) perceive blue and UV-A light (315–500 nm) [4, 5]; and UVR8 acts as the UV-B (~280 nm) photoreceptor [6]. In response to light, photoreceptors can directly act on numerous gene promoters throughout the genome to regulate the expression of their target genes in order for plants to rapidly adapt to their changing light environment [7–9]. In the absence of light, plants develop long hypocotyls, apical hook, unopened cotyledons and etioplastids, a unique developmental program known as skotomorphogenesis or etiolation. In the light, plants undergo photomorphogenesis, which is characterized by short hypocotyls, expanded cotyledons, and developed chloroplasts [1]. The skotomorphogenesis program is vital for terrestrial plants when their lives often start in the darkness of soil. The program prepares the plants for exposure to sunlight with vigor (a process known as greening), while inability to etiolate in darkness would be lethally damaged when exposed to light irradiation.

The *CONSTITUTIVELY PHOTOMORPHOGENIC 1 (COP1)* gene is essential for etiolation by acting as a repressor of photomorphogenesis, and its loss of function mutant display a constitutive photomorphogenic phenotype in darkness [10]. COP1 protein contains a RING finger, a coiled-coil domain, and WD-40 repeat domain, and it functions as an E3 ubiquitin ligase that targets a subset of photomorphogenic promoting factors for ubiquitination and degradation. In plant cells, COP1 exists as homodimers, which further stably associates with two SPA proteins, forming a tetrameric protein complex [11, 12]. Both COP1 dimerization and the interaction with SPA proteins are mediated through the coiled-coil domain of respective proteins. Association with SPA proteins enhances the activity of COP1 to targets substrate ubiquitination [12–14]. The substrates of COP1 in seedlings include LONG HYPOCOTYL (HY5), HY5 HOMOLOG (HYH), LONG HYPOCOTYL IN FAR-RED 1 (HFR1), LONG AFTER FAR-RED LIGHT 1 (LAF1), SALT TOLERANCE HOMOLOG 3 (STH3/BBX22) and PHYTOCHROME INTERACTING FACTOR 3-LIKE1 (PIL1) [14–20]. Besides seedling photomorphogenesis, COP1 also mediates the degradation of CONSTANS (CO), GIGANTEA (GI), EARLY FLOWERING 3 (ELF3), HYPERSENSITIVE RESPONSE TO TCV (HRT), SCAR1, GATA TRANSCRIPTION FACTOR 2 (GATA2) and MYC2, and plays critical roles in various developmental processes including flowering time, circadian clock, viral defense, root development, hormone signaling and controlling miRNA biogenesis [21–27]. COP1 is evolutionarily conserved from plants to animals. Mammalian COP1 has been reported to act as a tumor suppressor that targets oncoproteins c-Jun and ETS via its E3 ubiquitin ligase activity [28–31].

As a key regulator, COP1 protein level, activity, and localization are tightly controlled to ensure appropriate protein accumulation of its targets in response to developmental and environmental cues. In the dark, COP1 is enriched in the nucleus where it targets substrates for ubiquitination. Light triggers photoreceptors, including phyA, phyB, CRY1 and CRY2, to associates with SPA proteins or COP1, resulting in repression of the COP1-SPA E3 ubiquitin ligase activity [32–36]. This event is then followed by repartitioning of COP1 from the nucleus to the cytoplasm [37–40]. In addition, recent studies reveal that CSU1, SPAs and PIFs contribute to the modulation of COP1 protein level and activity as well [12,14, 41, 42].

In search of novel factors that modulate COP1 function or mediate its output, we have conducted a genetic screen for suppressors of *cop1-6*, a hypomorphic allele of *cop1* mutants [43]. This screen has previously led to successful identification of CSU1, an E3 ubiquitin ligase that targets COP1 [41]. Here we report another novel COP1 suppressor, designated as CSU2. Mutations in *CSU2* nearly completely suppress the constitutive photomorphogenic phenotype of *cop1-6* in darkness. CSU2 physically interacts and co-localizes with COP1 in nuclear speckles via a coiled-coil domain association. CSU2 is able to repress the COP1 E3 ubiquitin ligase activity. In addition, CSU2 has an important role in root development. Collectively, our genetic and biochemical data demonstrate that *Arabidopsis* CSU2 functions as a negative regulator of COP1, which serves to optimize the development of plants.

## Results

### Identification and molecular characterization of *csu2* via a *cop1-6* suppressor screen

**Fig 1:**
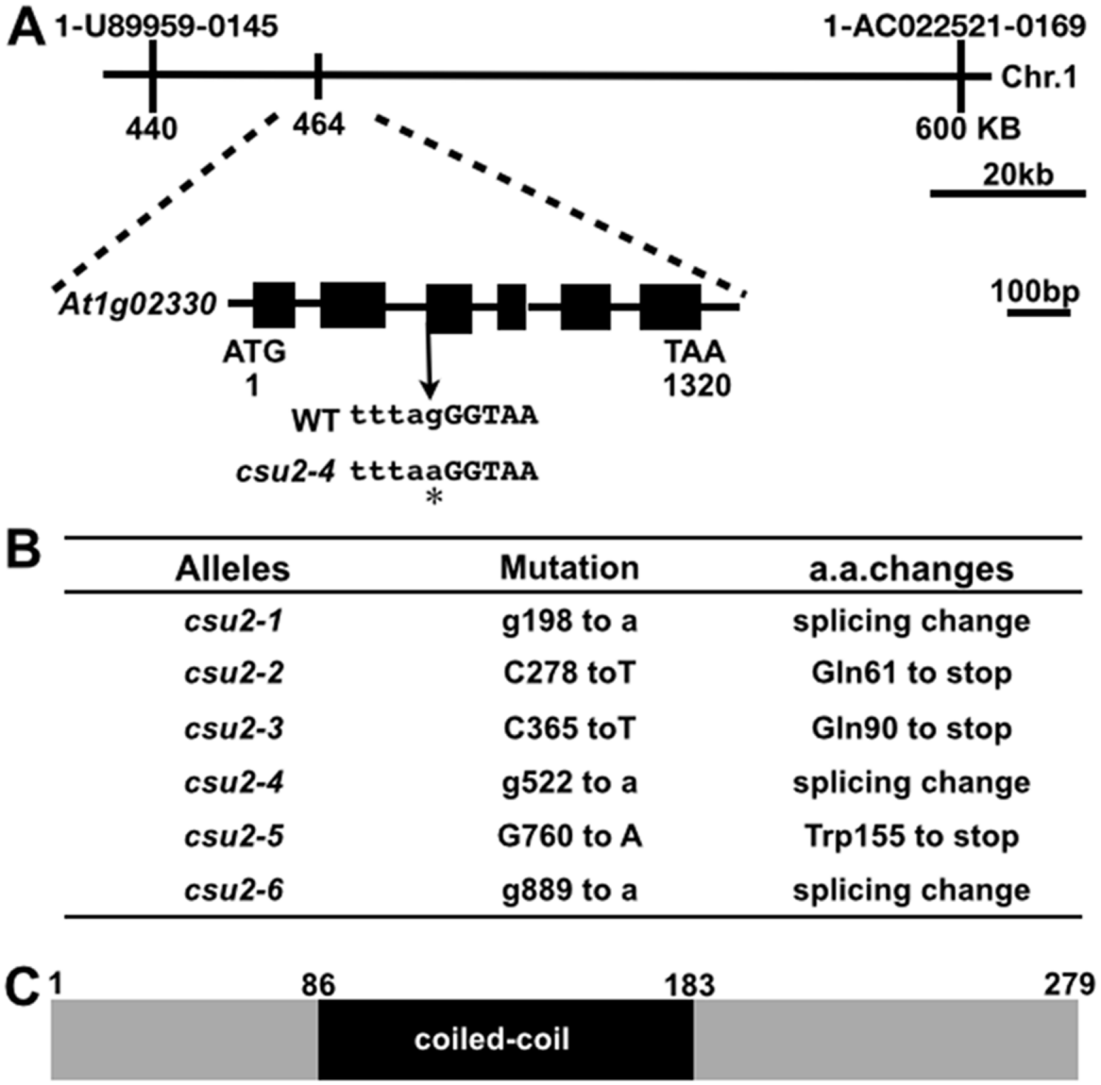
Map-based identification of *Arabidopsis CSU2*. (A) Map of the *CSU2* locus and gene structure. The exon is represented by a box, and the intron is represented by a line. The point mutation in *csu2-4* is marked with an asterisk. (B) Summary of mutations identified in the *csu2* alleles and the consequences of mutations to *CSU2* gene products. (C) CSU2 protein contains a coiled-coil domain. a.a., amino acids.

**Fig 2:**
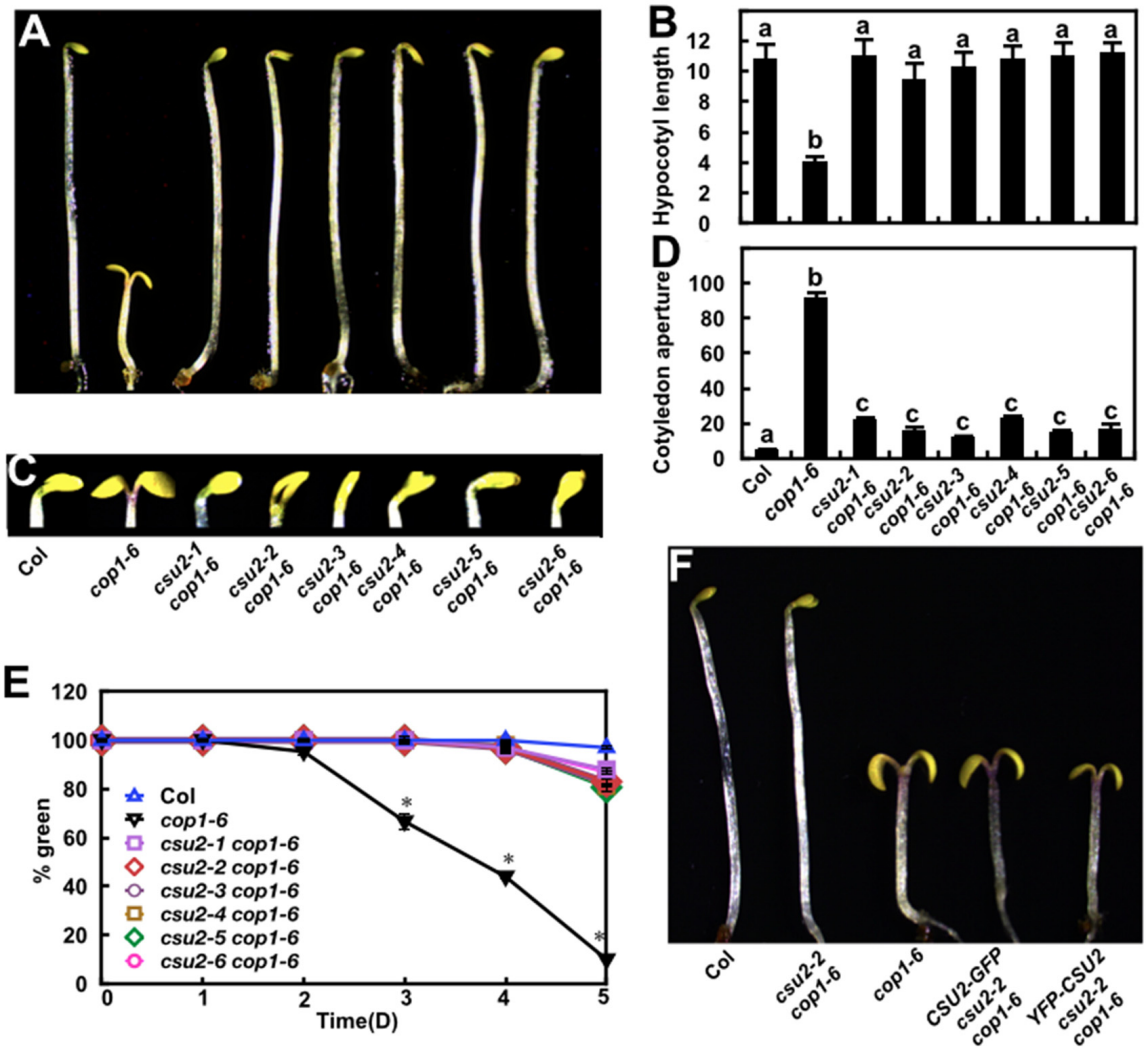
*csu2* suppresses *cop1*-6 in darkness. (A-B) Hypocotyl length (millimeter) of Col, *cop1-6* and *csu2 cop1-6* mutant seedlings grown in darkness for 5 days. Data are means ± SE; n ≥20. (C-D) Cotyledon phenotypes and separation angle of Col, *cop1-6* and *csu2 cop1-6* mutant seedlings grown in darkness for 5 days. The unit of cotyledon aperture is degree (°). Data are means ± SE; n ≥30. In panels (B) and (D), letters above the bars indicate significant differences as determined by one-way ANOVA with Tukey’s posthoc analysis (P <0.05). The experiment was repeated three times with similar results. (E) Quantification of photobleaching. Seedlings grown in the dark for indicated time periods were transferred to white light for 3 days. The number of green seedlings was counted against total seedlings, and percentages of green seedlings were presented. A total of 100 seedlings were used for each time point. Data are means ± SE; n = 3. Asterisks indicate statistical significance compared to *cop1-6* at the indicated times (one-way ANOVA with Tukey’s posthoc analysis, P <0.05). The experiment was repeated three times with similar results. (F) Complementation test. Morphology of Col, *csu2-2 cop1-6*, *cop1-6*, *CSU2-GFP csu2-2 cop1-6* and *YFP-CSU2 csu2-2 cop1-6* seedlings grown in darkness for 5 days.

A forward genetic screen was performed to explore *cop1* suppressors as described previously [41]. Six independent recessive alleles, located at a novel extragenic locus (*At1g02330*) named *cop1 suppressor* 2 (*csu2*), were recovered from this screen (Fig 1). Each of the mutant alleles (*csu2-1* to *csu2-6*) nearly completely suppressed *cop1-6* constitutive photomorphogenic phenotype in the dark (Fig 2). Since the mutation in *cop1-6* causes a splicing defect that leads to low expression of the *COP1* gene product [41, 43], we first tested whether mutations in *CSU2* affected *cop1-6* splicing profiles by a RNA pattern analysis. *csu2 cop1-6* produced five cryptically spliced profiles at intron 4 of *COP1*, similar to *cop1-6* (S1 Fig), suggesting that *csu2* suppressed *cop1-6* not by correcting its splicing defect. Thus, *csu2* was further characterized.

Via a combined chromosomal mapping and re-sequencing approach (see materials and methods for detail), we found that the *csu2-4* mutation changes the splicing junction “AG” at the 3' end of intron-2 to “AA”, thus disrupting the splicing principles of *CSU2*. Five additional mutant alleles from the same genetic complementation group were analyzed by PCR amplification followed by sequencing, which led to identification of distinct point mutation in each of the *csu2* mutant allele in *At1g02330* (Fig 1B). Thus, *At1g02330* defines the *CSU2* gene.

*CSU2* is a single-copy gene encoding a predicted 279 amino acid protein in *Arabidopsis* (Fig 1C). Only one putative domain, a coiled-coil domain, was identified in CSU2. CSU2 is evolutionarily conserved. The amino acid sequence identity of *Arabidopsis* CSU2 to its orthologs from *Homo sapiens*, *Mus musculus*, *Danio rerio*, *Drosophila melanogaster*, and *Oryza saliva* is *34%*, *34%*, *35%*, *40%* and *61%* respectively, with the coiled-coil domain being the most conserved region (S2 Fig).

### Suppression of *cop1*-6 phenotype by *csu2* mutations

*cop1-6* mutant is unable to etiolate in darkness [43], and is defective in greening upon transfer to white light [44]. Mutations in *CSU2* almost completely restored *cop1-6* constitutive photomorphogenic phenotype to WT phenotype in the dark (Fig 2). Hypocotyl length of all six different *csu2 cop1-6* mutant lines was essentially indistinguishable from that of WT seedlings (Fig 2A and 2B). Although the cotyledons of *csu2 cop1-6* were slightly open, the cotyledon apertures of all six independent *csu2 cop1-6* mutant lines were significantly smaller than that of *cop1-6* (Fig 2C and 2D). Moreover, although most dark-grown *cop1-6* seedlings were unable to green when transferred from dark to white light, the greening rate of etiolated *csu2 cop1-6* mutant seedlings was restored to a level comparable to that of WT (Fig 2E).

To verify that the suppression of the *cop1-6* phenotype in *csu2 cop1-6* etiolated seedling was indeed caused by the mutation in *CSU2* gene only, we introduced *CSU2-GFP* and *YFP-CSU2* into the *csu2-2 cop1-6* double mutant. Consistently, *CSU2-GFP csu2-2 cop1-6* and *YFP-CSU2 csu2-2 cop1-6* transgenic seedlings displayed constitutive photomorphogenic phenotype similar to that of *cop1-6* single mutant in the dark, indicating that a functional CSU2 could complement the phenotype conferred by *csu2-2* in *cop1-6* background in darkness (Fig 2F).

Not only did *csu2* rescue the dark phenotype of *cop1-6*, *csu2* also partially suppressed the short hypocotyl phenotype of *cop1-6* seedlings grown under various light conditions tested (white, red, far-red and blue) (S3 Fig). The dwarf phenotype of *cop1-6* adult plants under the long-day condition (16 h light / 8 h dark) for 30 days was also partially suppressed by *csu2* (S4 Fig). All together, these genetic data suggest that *csu2* almost completely suppress *cop1-6* in the dark and partially in the light.

### The *csu2* mutations alone are hyposensitive to white light

**Fig 3:**
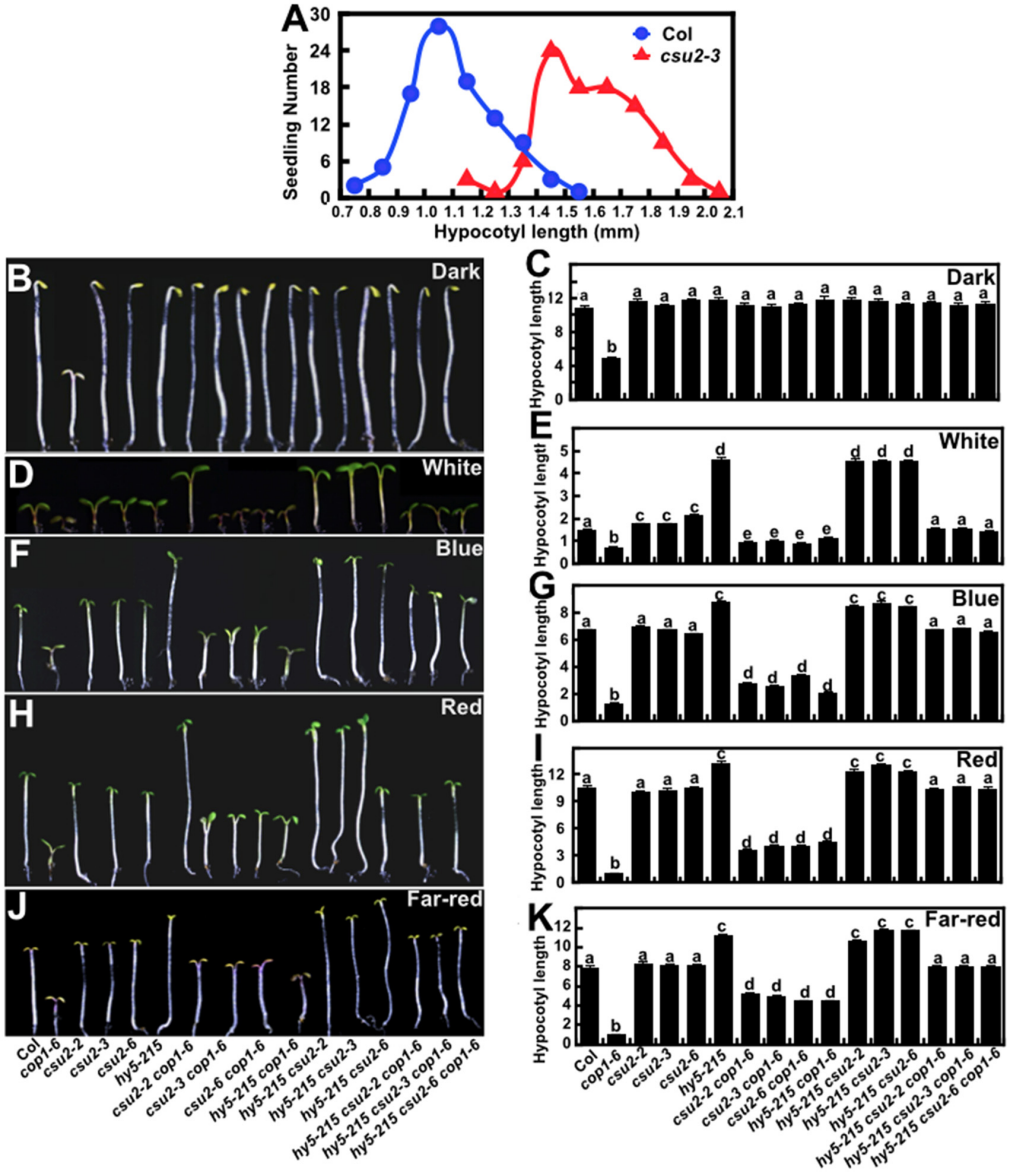
*hy5* together with *csu2* co-suppress the short hypocotyl phenotype of *cop1-6*. (A) Distribution graph showing the differences in hypocotyl length between Col and *csu2-3* seedlings grown in constant white light (112.3 μmol/m^2^/s) for 5 days. At least 80 seedlings were measured for hypocotyl length each time. The experiments were performed 3 times with similar results. (B-K) Hypocotyl phenotype and length (millimeter) of Col and various mutants grown in the dark (B-C), white (112.3 μmol/m^2^/s) (D-E), blue (0.62 μmol/m^2^/s) (F-G), red (6.78 μmol/m^2^/s) (H-I) and far-red (1.46 μmol/m^2^/s) (J-K) and for 5 days. Data are means ± SE; n≥20. Letters above the bars indicate significant differences as determined by one-way ANOVA with Tukey’s posthoc analysis (P<0.05). The experiment was repeated three times with similar results.

To examine whether mutations in *CSU2* have defect in light responses by themselves, single mutants of all six alleles (*csu2-1* to *csu2-6*) were isolated from the F2 generation of *csu2 cop1-6* crossed with Col and grown under various light conditions (dark, white, blue, red and far-red) for five days. At low fluence rate of white light (15.7 μmol/m^2^/s), the hypocotyl length of *csu2* mutant seedlings was indistinguishable from that of WT (S5 Fig). At the higher fluence of white light (33.3 and most evidently 112.5 μmol/m^2^/s), all six independent *csu2* single mutants displayed statistically significantly longer hypocotyls than did WT seedlings (Fig 3 and S5 Fig). However, *csu2* mutant seedlings did not differ significantly from WT seedlings under all monochromatic light (blue, red and far-red) conditions tested (S6 Fig). The fact that *csu2* mutant seedlings were specifically hyposensitive to higher fluence rate of white light suggests that CSU2 acts as a positive regulator in the high fluence white light induced inhibition of hypocotyl elongation.

HY5 transcription factor is a major downstream effector of COP1, whose mutation can also suppress *cop1-6* [15, 44, 45]. The hypocotyl length of *hy5-215 csu2* double mutant seedlings was similar to that of *hy5-215* single mutants in all light conditions tested including high fluence of white light, in which *csu2* exhibited longer hypocotyls than WT (Fig 3B–3K). This result indicates that *hy5-215* is epistatic to *csu2* in the control of hypocotyl growth. Although either *csu2* or *hy5* alone only partially suppressed *cop1-6* in the light, both mutations together (*hy5 csu2 cop1-6*) restored *cop1-6*’s hypocotyl length to that of WT seedlings under all light conditions tested (white, blue, red and far-red) (Fig 3B–3K). It appeared that *CSU2* and *HY5* act additively in the suppression of *cop1* hypocotyl phenotype in the light. We suggest from these genetic data that *CSU2* and *HY5* work independently and additively, with *HY5* acting downstream of *CSU2*, to counter *COP1*’s action in the control of hypocotyl elongation.

### CSU2 interacts with COP1 through their coiled-coil domain association

**Fig 4:**
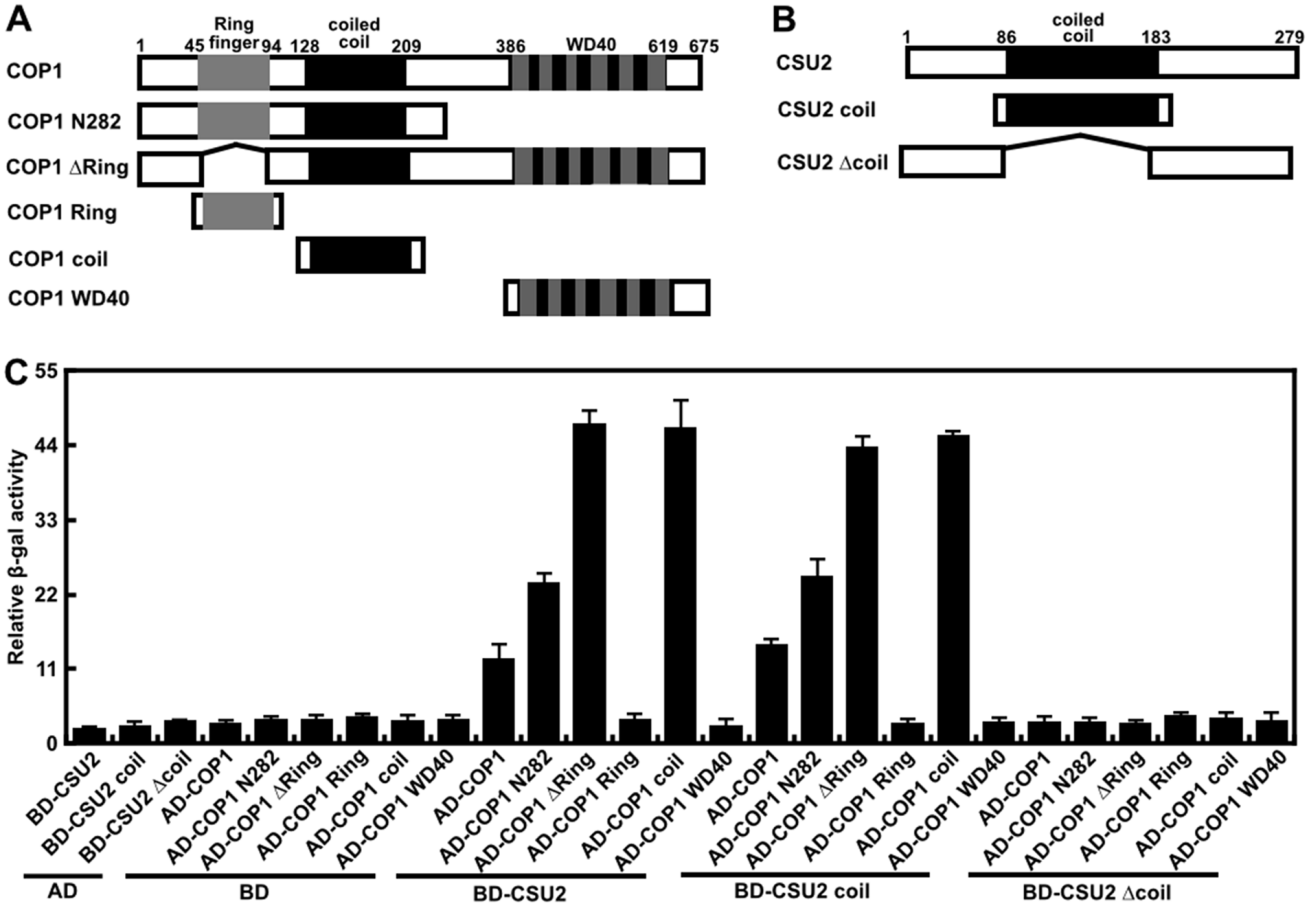
CSU2 interacts with COP1 in yeast, and their coiled-coil domains are required for the interaction. (A) Schemes of the domain structure of COP1 and the truncated COP1 proteins. (B) Schemes of the domain structure of CSU2 and the truncated CSU2 proteins. (C) Yeast two-hybrid interactions between the indicated CSU2 and COP1 proteins. Data are means ± SD; n = 5.

To understand the mechanism of CSU2, we examined a possible protein-protein interaction between CSU2 and COP1 by a yeast-two-hybrid assay. As shown in Fig 4, CSU2-COP1 interaction was evident as indicated by increased β-galactosidase activity compared to BD-CSU2 and AD-COP1 alone. COP1 possesses three protein-protein interaction domains, Ring-finger, coiled-coil and WD40 domains, while CSU2 contains only one predictable coiled-coil domain. To identify which COP1 domain is responsible for the interaction with CSU2, a deletion analysis of the COP1 fragment was carried out. Interestingly, COP1 N282, COP1 Δring and COP1 coil containing the COP1 coiled-coil domain, showed even stronger interaction with CSU2 than full-length COP1 (Fig 4). In contrast, COP1 Ring and COP1 WD40, which lack COP1 coiled-coil domain, were unable to interact with CSU2. Thus, the coiled-coil domain of COP1 is necessary and sufficient for interaction with CSU2. Next, we examined whether the coiled-coil domain of CSU2 was sufficient for the CSU2-COP1 interaction. Similar to the full-length CSU2, the CSU2 coil domain was capable of interacting with COP1, COP1 N282, COP1 Δring and COP1 coil, but not COP1 Ring and COP1WD40. In addition, CSU2 Δcoil, which lacks the coiled-coil domain, was unable to interact with full-length COP1 or any of the COP1 deletion constructs (Fig 4). Taken together, those data indicates that CSU2 interacts with COP1 through their respective coiled-coil domains.

**Fig 5:**
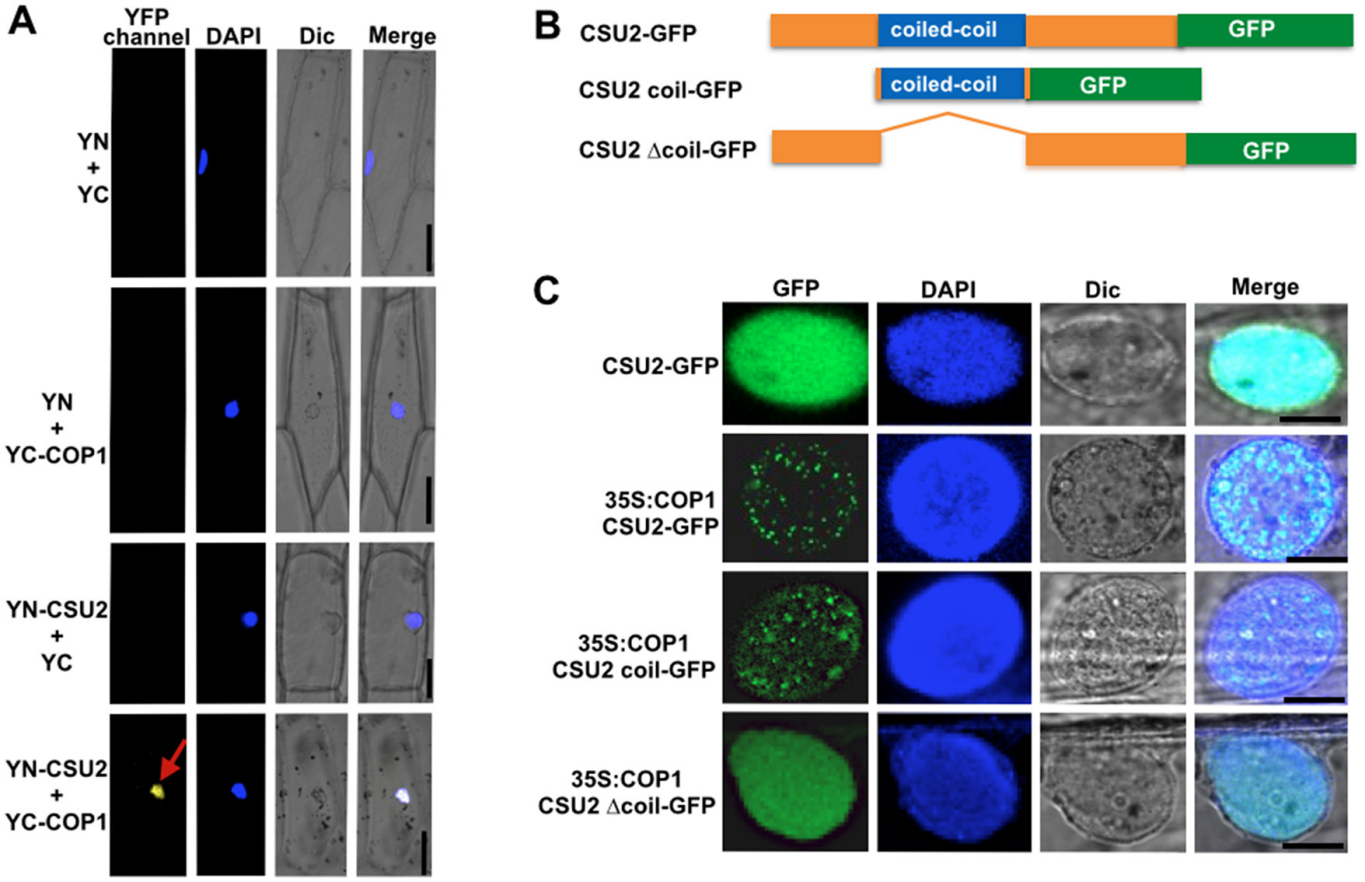
CSU2 interacts and colocalizes with COP1 in living plant cells. (A) BiFC assay showing the interaction of CSU2 with COP1 in oion epidemal cells. Full-length CSU2 and COP1 were fused to the split N- or C-terminal (YN or YC) fragments of YFP. Unfused YFP N-terminal (YN) or YFP **C**-terminal (YC) fragments were used as negative controls. DAPI staining marked the positions of nuclei; Dic, differential interference contrast in light microscope mode; Merge, merged images of YFP channel, DAPI and Dic. Red arrow indicates the position of YFP speckle. Bar = 100 μm. (B) Schemes of the CSU2-GFP, CSU2 coil-GFP and CSU2 Δcoil-GFP constructs. (C) Nucleus of a cell co-expressing 35S:COP1 (untagged) with CSU2-GFP, CSU2 coil-GFP or CSU2 Δcoil-GFP. GFP channel, GFP channel image; DAPI, nucleus marker; Dic, differential interference contrast in light microscope mode; Merge, merged images of GFP, DAPI and Dic. Bar = 20 μm.

We next performed the Bimolecular Fluorescence Complementation Assays (BiFC). Constructs of CSU2 fused with N-terminal of YFP (YN-CSU2) and COP1 fused with C-terminal of YFP (YC-COP1) were generated. When YN-CSU2 and YC-COP1 were co-transformed into onion (*Allium cepa*) epidermal cells, strong YFP fluorescence signals were observed in the nucleus, indicating that CSU2 can interact with COP1 (Fig 5A). Furthermore, we examined whether Fluorescence Resonance Energy Transfer (FRET) could occur between the two fusion proteins CFP-CSU2 and YFP-COP1 using the acceptor photobleaching technique. Here, we co-expressed CFP-CSU2 with YFP-COP1 in onion epidermal cells and excited them with 405- and 514-nm wave lengths light sources. Both CFP and YFP fluorescence were detected before bleaching. CFP-CSU2 produced uniform fluorescence throughout the nucleus, while YFP-COP1 formed nuclear speckles (S7A and S7B Fig). Since FRET occurs only at nanometer scale distances [46], only YFP-COP1 speckles areas were chosen for bleaching by 514-nm laser. After bleach, emission of YFP-COP1 was reduced dramatically, whereas emission from CFP-CSU2 in the region of interest increased (S7A and S7B Fig), indicating that FRET had occurred between the two proteins prior to the bleach. As a control, we did not detect FRET between YFP and CFP-CSU2 (S7C and S7D Fig). Together, these data support a conclusion that the CSU2 interacts with COP1 in living plant cells.

### CSU2 is a nuclear protein and co-localizes with COP1 in plant cells

COP1 forms nuclear speckles in darkness and is able to recruit several interacting proteins to those loci [14, 16, 45, 47]. Our FRET assay data indicated that COP1 and CSU2 might co-localize in the nuclear speckles (S7A Fig). To further substantiate this finding, we performed transient co-localization assays using GFP tagged CSU2 fusion protein in onion epidermal cells (Fig 5B and 5C). Unlike COP1, CSU2 localized uniformly throughout the nucleus (Fig 5C). However when we co-expressed COP1 (35S:COP1) together with CSU2-GFP, we detected consistent nuclear speckles (Fig 5C). Since CSU2-GFP by itself only produces a uniform fluorescence, the observation of nuclear speckles when co-expressed with untagged COP1 suggests that CSU2 is recruited into nuclear speckles by COP1. Moreover, untagged COP1 (35S:COP1) could confer nuclear speckle formation to a co-expressing CSU2 coil-GFP but not CSU2 Δcoil-GFP (Fig 5C). These observations provide further evidence that interaction of COP1, via the coiled-coil domain of CSU2, is required and sufficient for recruitment of CSU2 into the nuclear speckles in living plant cells.

To determine whether CSU2 is a nuclear protein *in planta*, we examined its localization pattern in *35S*:*CSU2-GFP csu2-2* transgenic *Arabidopsis* seedlings where CSU2-GFP has been shown to be functional (Fig 2F). As shown in S8 Fig, CSU2-GFP was found within the nucleus both in darkness and light, confirming that CSU2 is a nuclear protein *in planta*.

### CSU2 represses the COP1 E3 ubiquitin ligase activity

COP1 targets a group of interacting proteins for ubiquitination and degradation. Therefore, we investigated whether COP1 regulates CSU2 abundance. YFP fluorescence signal intensity was comparable in the *YFP-CSU2 csu2-2* and *YFP-CSU2 csu2-2 cop1-6* transgenic seedlings (S9 Fig). In addition, similar protein levels of YFP-CSU2 were detected in these two transgenic lines grown in various light conditions tested (dark, white, blue red and far-red) (S10 Fig). These findings suggest that COP1 does not regulate CSU2 abundance.

**Fig 6:**
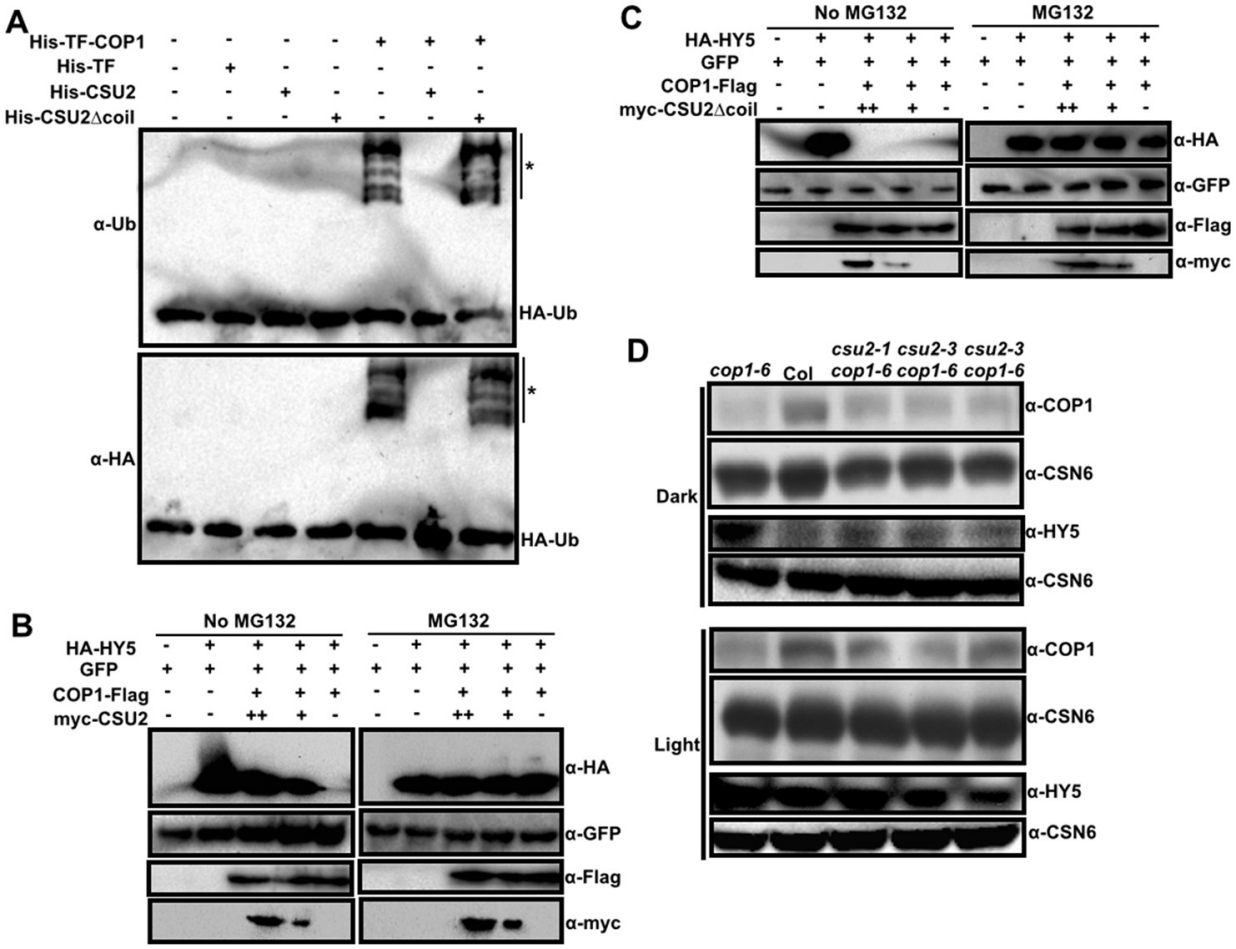
CSU2 represses the COP1 E3 ubiquitin ligase activity. (A) Repression of COP1 ubiquitination activity by CSU2. Ubiquitination assays were performed in a reaction mix containing UBE1(E1), UbcH5b (E2), and HA-tagged ubiquitin (HA-Ub). Recombinant 6×His-TF (500 ng), 6×His-CSU2 (500 ng), 6×His-CSU2 Δcoil (500 ng), and 6×His-TF-COP1 (500 ng) were added in the reactions as indicated. Asterisks indicate ubiquitinated His-TF-COP1 detected by ubiquitin and HA antibodies, respectively. TF represents Trigger Factor. (B-C) *In vitro* degradation of HY5 in mixed lysates from transiently infected *Nicotiana benthamiana* leaves that separately expressing HA-HY5, GFP, COP1-Flag, myc-CSU2 or myc-CSU2 Δcoil. The HA-HY5 extract was mixed with GFP and COP1-Flag extracts containing either myc-CSU2 (B) or myc-CSU2 Δcoil (C) extracts without (left) or with (right) 50 μM MG132. HA-HY5, GFP, COP1-Flag and myc-CSU2 or myc-CSU2 Δcoil proteins were immunoblotted with HA, GFP, Flag and myc antibodies, respectively. “-”, “+” and “++”indicate 0 μg, 100 μg and 200 μg corresponding protein extracts were used in degradation and immunoblotting analysis. (D) Steady state protein levels of COP1 and HY5 in Col, *cop1-6* and *csu2 cop1-6* seedlings grown in the dark or white light for five days as detected by COP1 and HY5 antibodies, respectively.

The coiled-coil domain of COP1 is necessary for its dimerization [13] and for interacting with SPA proteins [11,12,48]. These interactions enhance COP1’s E3 ubiquitin ligase activity [12, 14]. Given that CSU2-COP1 association is through COP1 coiled-coil domain, we wanted to test whether CSU2 can affect COP1 activity. Consistent with previously described *in vitro* ubiquitination assay [14, 17], we detected a robust COP1 dependent ubiquitination activity, and this activity was drastically inhibited when CSU2 was present in the reaction (Fig 6A). Remarkably, COP1’s ubiquitination activity was not affect by CSU2 Δcoil, which lacks coiled-coil domain (Fig 6A). Therefore CSU2 can inhibit COP1 E3 activity *in vitro*, and the inhibition is dependent on CSU2’s COP1-binding domain.

HY5 is a major ubiquitination substrate of COP1 in seedlings, and its level of accumulation correlates with seedling photomorphogenesis [15, 45]. To examine the effect of CSU2 on COP1’s activity toward a specific substrate, we performed a cell-free HY5 degradation assay in cell lysates, in which degradation of HY5 was dependent on the presence of COP1 (Fig 6B and 6C). Notably, with decreasing amounts of CSU2 in the mixture, the protein level of HY5 also decreased (Fig 6B). In contrast to full length CSU2, CSU2 Δcoil had no effect on COP1 mediated degradation of HY5 (Fig 6C). As a validation of the assay, degradation of HY5 protein could be blocked by proteasome inhibitor MG132 treatment. The GFP protein, as an internal control, remained relatively stable under all the tested conditions (Fig 6B and 6C). Together, these data show that CSU2 represses the COP1 ubiquitination activity *in vitro*, and repress COP1-dependent degradation of HY5 in a cell-free degradation assay. In both cases, the coiled-coil domain of CSU2 is required for the repression of COP1 activity.

### *csu2* mutations allow seedlings with low COP1 level to keep HY5 protein at minimum in the dark

Prompt by CSU2’s activity in repressing COP1’s E3 ubiquitin activity *in vitro*, and in inhibiting HY5 degradation in the cell-free assay, we determined the steady state levels of COP1 and HY5 proteins in the seedlings of *csu2 cop1-6* compared to *cop1-6*, and wild type (Fig 6D). The levels of COP1 in *csu2 cop1-6* appeared slightly higher than that of *cop1-6*, but still substantially lower than WT in both dark- and light-grown seedlings (Fig 6D). The reason of the slight increase of COP1 is discussed later. The important point is that, even with clearly reduced amount of COP1, the dark-grown *csu2 cop1-6* seedlings nevertheless managed to keep HY5 protein level as low as in WT, which was drastically decreased compared to *cop1-6* (Fig 6D). Presumably, despite of reduced level of COP1 in *csu2 cop1-6*, but due to lack of CSU2-mediated inhibition, the total activity of COP1 seems sufficient to prevent HY5 accumulation in the dark. The slight increase of COP1 level in *csu2 cop1-6* might also have contributed to the suppression of HY5 in the dark.

We next asked whether *csu2* mutant seedlings display altered protein accumulation of additional components of light signaling. Under both dark and light conditions, phyA, phyB, COP1, HY5 and SPA1-4 (dark only) accumulated at comparable levels in WT and *csu2* mutant seedlings (S11 Fig). Thus we have not detected an effect of CSU2 on protein abundance of these light-signaling components under normal growth conditions.

### CSU2 mediates the primary root growth in response to light

**Fig 7:**
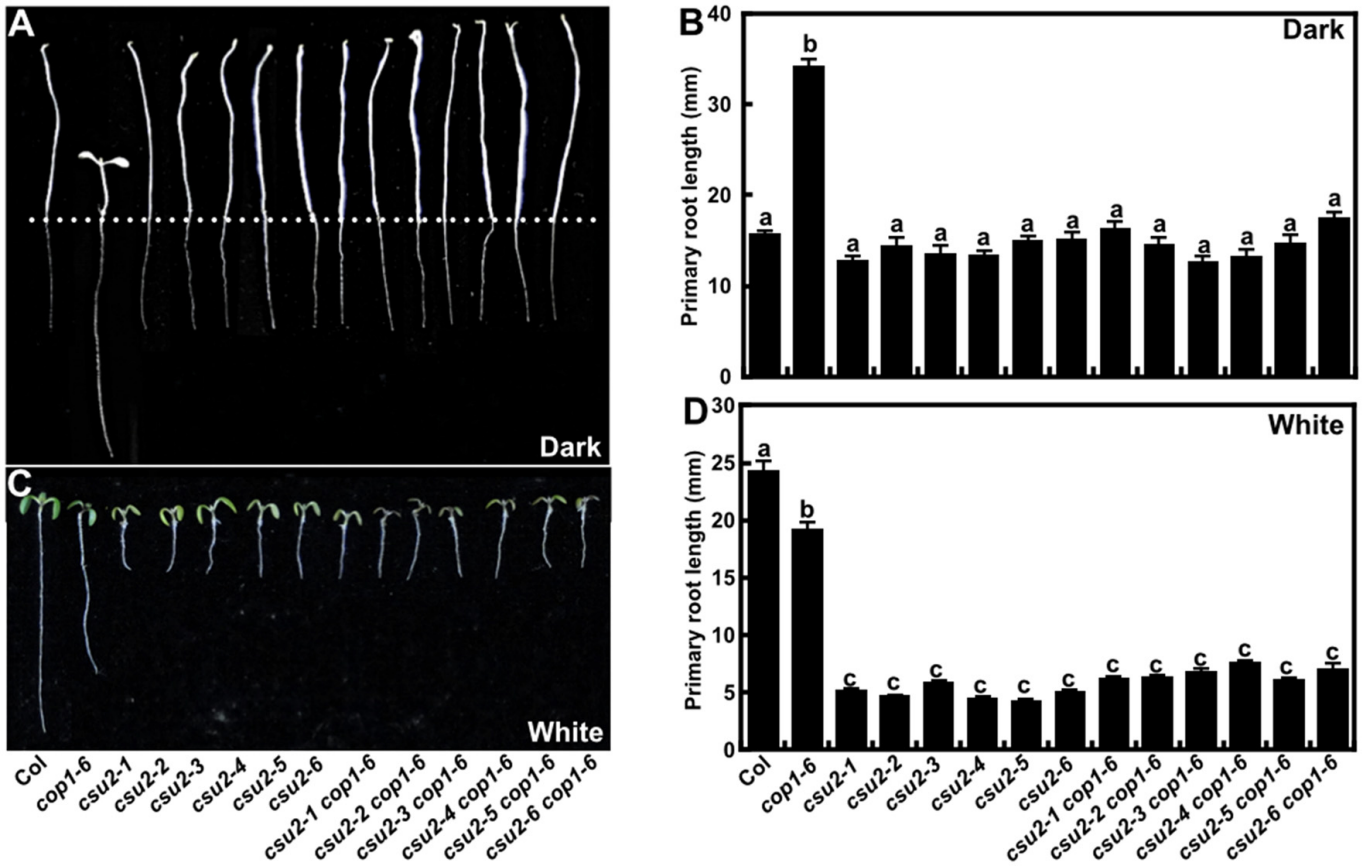
*csu2* mutants display reduced primary root length in the light. (A-B) Root phenotype and length of Col, *csu2*, and *csu2-2 cop1-6* cultivated on vertical plates for 5 days in the dark. Dotted line indicates the shoot-root junction. Data are means ± SE; n≥20. (C-D) Root phenotype and length of Col, *csu2* and *csu2 cop1-6* cultivated on vertical plates for 5 days under continuous white light conditions. Data are means ± SE; n≥20. In panels (B) and (D), letters above the bars indicate significant differences as determined by one-way ANOVA with Tukey’s posthoc analysis (P<0.05). The experiment was repeated three times with similar results.

Light-grown seedlings display longer primary roots than etiolate seedlings, and *cop1* mutant seedlings display an opposite root growth pattern [49]. To investigate the role of CSU2 in the root development, the six different *csu2* mutant lines were germinated on vertical plates and grown for five days under dark or constant white light conditions. In the dark, *cop1-6* displayed longer roots than did WT, while *csu2* displayed the same root length to that of WT. *csu2 cop1-6* double mutants exhibited roots similar to those of *csu2* or WT seedlings, indicating that the long root phenotype of *cop1-6* was completely suppressed by *csu2* (Fig 7A and 7B). In the light however, all six different *csu2* single mutants displayed dramatically shorter roots than did WT or *cop1-6* (Fig 7C and 7D), and *csu2 cop1-6* showed similar root length as *csu2* single mutants. To further confirm that the short primary root phenotype is caused by disruption of CSU2, we investigated the primary root phenotypes of *35S*:*myc-CSU2 csu2-2* as well as *35S*:*CSU2-GFP csu2-2* and *35S*:*YFP-CSU2 csu2-2* transgenic lines (S9 Fig). In all cases, expression of *CSU2* transgene rescued the shortened primary root phenotype of *csu2-2* (S9 Fig), indicating the short primary root phenotype is resulted from lack of a functional CSU2. Taken together, these findings show that *csu2* completely suppresses *cop1* long primary root phenotype in the dark, that CSU2 is required for light stimulated primary root development, and that *csu2* is epistatic to *cop1* with respect to the primary root phenotype in the light.

**Fig 8:**
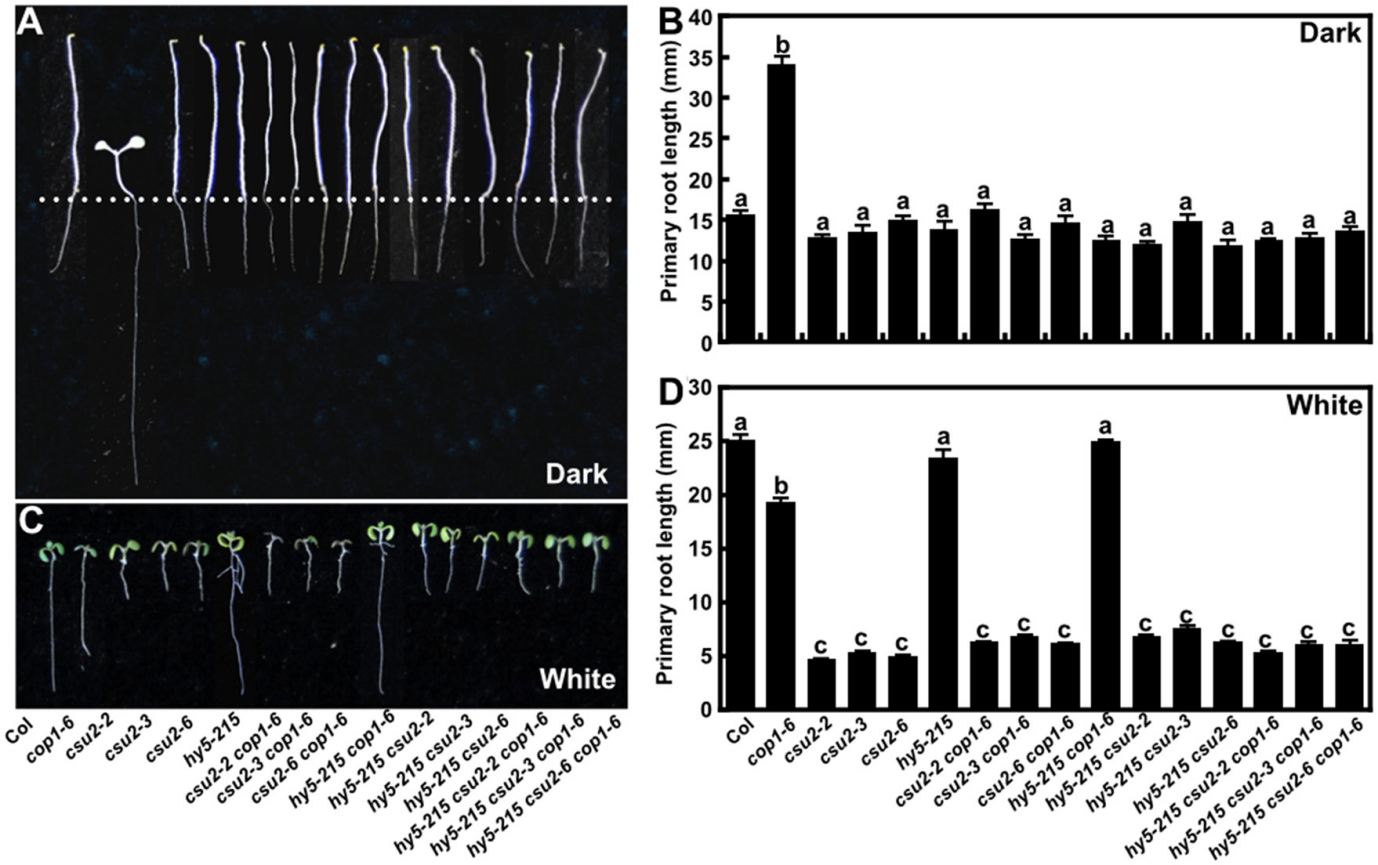
*csu2* is epistatic to *cop1* or *hy5* in the root phenotype. (A-B) Root phenotype and length of Col and various mutants cultivated on vertical plates for 5 days in the dark. Dotted line indicates the shoot-root junction. Data are means ± SD; n≥20. (C-D) Root phenotype and length of Col and various mutants cultivated on vertical plates for 5 days under continuous white light conditions. Data are means ± SD; n≥20. In panels (B) and (D), letters above the bars indicate significant differences as determined by one-way ANOVA with Tukey’s posthoc analysis (P<0.05). The experiment was repeated three times with similar results.

To further investigate the genetic relationship among *csu2*, *hy5* and *cop1* with respect to root phenotypes, we studied the *hy5 csu2*, *hy5 cop1* and *hy5 csu2 cop1* double and triple mutants. In the dark, all the double and triple mutants exhibited root phenotypes similar to those of WT (Fig 8A and 8B). Under white light condition, the root length of *hy5 csu2*, or *hy5 csu2 cop1* double and triple mutant seedlings resembled *csu2* short roots phenotype (Fig 8C and 8D), suggesting a different genetic relationship of those three loci in mediating light regulation of root development and in hypocotyl growth. With regard to primary root growth, the requirement for functional CSU2 overrides the regulatory functions of COP1 and HY5.

## Discussion

COP1 is a central player of light regulated developmental processes. The mechanism of COP1 in the regulation of these processes is by working as an E3 ubiquitin ligase that targets an array of important gene expression regulators for proteolysis in a manner that is dependent on developmental stages and/or environmental cues [23, 50, 51]. Using seedling photomorphogenesis as a model, we have isolated six different alleles of *csu2* mutants, each of which can completely suppress *cop1-6* phenotype and restore etiolation when grown in the dark. In this system, the extent of photomorphogenic development of seedlings correlates quantitatively with HY5 protein abundance *in planta*, and HY5 protein levels normally correlates inversely with the nuclear abundance of COP1 [15]. Here we report that CSU2 interacts and co-localizes with COP1 in the plant cells, and it negatively regulates COP1 E3 ubiquitin ligase activity, which directly affects HY5 stability. Thus, CSU2 functions as a repressor of COP1 to regulate aspects of plant development.

### Mechanism of suppression of COP1 by CSU2

**Fig 9:**
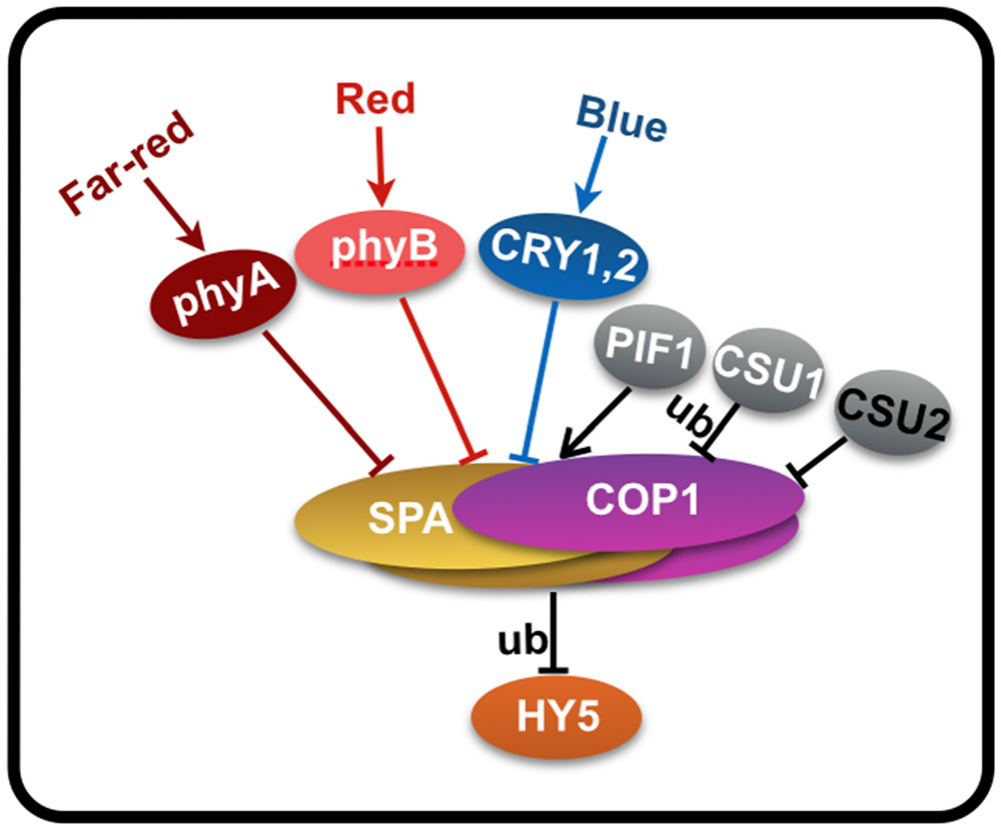
A simplified model showing regulation of COP1 by CSU2 as well as other factors. Two COP1 and two SPA proteins form stable complex and targets downstream substrates HY5 for ubiquitination and degradation to repress photomorphogenesis. In response to light, phyA, phyB, CRY1 and CRY2 disrupt the formation of COP1-SPA complex to repress its E3 activity. PIF1 interacts with COP1-SPA complex to enhance its activity. CSU1 targets COP1 for ubiquitination in maintaining its homeostasis, and CSU2 binds to COP1 through their respective coiled-coil domains to repress its activity. These factors work in concert to regulate COP1 level and activity. HY5 represents a downstream effector and functional output of COP1 in seedling photomorphogenesis. This model is also support by the genetic analysis *hy5*, *csu2* and *cop1* (Fig 3).

COP1 is regulated in a number of different ways. Not only is COP1 nucleocytoplasmic partitioning regulated by light, low temperature, heat shock and ethylene [37, 52–54], its protein abundance is regulated by CSU1, an E3 ubiquitin ligase identified by the same screen as CSU2 [41] (Fig 9). COP1 activity is rigorously regulated as well. It has been demonstrated that PIFs and SPAs interact with COP1, and enhance COP1 ubiquitylation activity [12, 14, 42], while photoreceptor activation inhibits COP1 E3 activity [32–36] (Fig 9).

In etiolated seedlings, two SPA proteins associate with COP1 homo-dimers and form stable core complexes through their respective coiled-coil domains, which in turn, serve to enhance the COP1 activity possibly by increasing substrate recruitment [11, 12, 14]. Upon exposure to light, phyA, phyB and CRY1 interact with SPA, while the CRY2 binds to COP1. These interactions result in destabilization and disruption of the COP1-SPA complex, and consequently inhibition of COP1 E3 ubiquitin ligase activity [32–36]. In a similar fashion, we speculate that CSU2 mediated repression may also be directed at dismantling COP1-SPA complex and/or blocking COP1 dimerization.

CSU2 and COP1 interact through their coiled-coil domains, and CSU2 coiled-coil domain is necessary for the repression of COP1 activity *in vitro* (Figs 4, 5 and 6). Moreover, CSU2 can inhibit COP1-mediated HY5 turnover in a cell-free plant extract assay, also in a coiled-coil domain dependent manner. The coiled-coil domain of COP1 is responsible for its self-dimerization, a necessary conformation for its E3 ubiquitin ligase activity [13]. Thus it is possible that CSU2-COP1 association may interfere with the COP1 self-dimerization (*in vitro*) as well as COP1-SPA interaction (*in vivo*), which may result in destabilization COP1 dimer and COP1-SPA complexes, in a similar mechanism to activated photoreceptors.

We found that *csu2 cop1-6* seedlings contained slightly higher amount of COP1 protein than *cop1-6* alone, although still substantially lower than in wild type (Fig 6D). This could also be explained by above mentioned hypothesis: lack of CSU2’s competitive binding to COP1 coiled-coil domain would stabilizes COP1 dimerization and COP1-SPA complex, which would protect COP1 protein to certain extent. Nonetheless the slight increase of COP1 protein alone cannot fully account for the complete suppression of HY5 level in *cop1-6 csu2* double mutants in the dark (Fig 6D). We postulate that both stabilization of COP1, and more importantly an increase of COP1 activity, occur in the absence of CSU2, which most likely underlie the mechanism of suppression of *cop1-6* by *csu2*.

### *csu2* is an allele-specific suppressor of *cop1-6*

*csu2* specifically suppresses the *cop1-6* allele, but not *cop1-1* and *cop1-4* (Fig 2, S13A and S13B Fig). In *cop1-6*, the mutation causes a splicing defect that eventually produces COP1-6 mutant protein with five additional amino acids insertion at severely decreased level [43]. COP1-6 protein is largely biologically functional [41]. The strong allele *cop1-1* has a 66-bp deletion, causing a deletion from amino acid 355 to 376 (~74 kD) [43]. The COP1-1 protein is produced to wild-type levels (S13C Fig), but is severely functionally defective, as indicated from the mutant phenotype. *cop1-4* mutant accumulates a truncated COP1 protein (~33 kD) containing only the N-terminal 282 amino acids, and it is expressed at same or reduced level compared to wild type [43] (S13C Fig). Interestingly, when COP1-4 (N282) protein is overexpressed, it can cause a dominant negative phenotype in wild type background [55]. Thus the loss-of-function mechanism of *cop1-4* mutation is rather complicated. Among the three *cop1* mutant alleles, *cop1-6* is the only hypomorphic allele, as it produces a functional protein at a lower level. Since *csu2* suppression works by releasing the repression on a functional COP1 protein, only *cop1-6* can be effectively suppressed by lack of CSU2. The failure of suppression of *cop1-1* and *cop1-4* by *csu2* may primarily attribute to the nature of COP1-1 and COP1-4 mutant gene products, which are functionally defective. It should be mentioned that *hy5* is able to partially suppress *cop1-1* and *cop1-4*, as well as *cop1-6* [44], because HY5 is a downstream factor that mediates COP1’s output.

### The regulatory hierarchy of CSU2-COP1 pair is tissue-dependent

*Arabidopsis* exhibited longer roots in the light and shorter roots in darkness, while *cop1-6* displayed a revered phenotype [49]. In the dark, COP1 directly targets SCAR1, a positive regulator of root growth for ubiquitination and protein turnover [25], which contributes to the longer primary root phenotype of *cop1* in darkness. The drastic long primary root length of *cop1-6* grown in darkness was completely suppressed by *csu2* (Fig 7). In the light however, both CSU2 and COP1 function as positive regulators of root development. The *csu2* mutant seedlings developed severely shortened roots in the light, suggesting CSU2 is required for primary root growth in response to light (Fig 7).

Our study revealed that CSU2 may act upstream of COP1 in the hypocotyls, whereas may genetically act downstream of COP1 in the roots, and that a functional CSU2 protein is required for primary root growth both in WT and in *cop1*-6 (Fig 8). Thus, it appears that different regulatory module of CSU2-COP1 pair may exist in the hypocotyl and root cells. Nevertheless, the exact functional relationship between COP1 and CSU2 in regulation of root growth needs further investigation.

## Materials and Methods

### Plant materials and growth conditions

The *cop1-6* [43], *hy5-215* [44], *csu2 cop1-6* (*csu2-1 cop1-6* to *csu2-6 cop1-6*), and *csu2* (*csu2-1* to *csu2-6*) (this study) mutants are in the Columbia-0 (Col-0) ecotype. Seeds were surface sterilized with 30% commercial Clorox bleach and 0.02% Triton X-100 for ten min and washed three times with sterile water, and sown on 1×Murashige and Skoog (MS) medium supplemented with 0.4% Bacto-agar (Difco) and 1% sucrose. The seeds were stratified in darkness for three days at 4°C, and then transferred to light chambers maintained at 22°C.

### Genetics screen, identification and characterization of *CSU2*

The genetics screen, identification and characterization were previously described [41]. Genetic complementation tests showed that six different *csu2* (*csu2-1 cop1-6* to *csu2-6 cop1-6* lines) EMS mutations were allelic to each other. Homozygous mutant suppressor plants were crossed to wild-type plants (Col-0), and segregation in the F2 generations was analyzed in the dark to distinguish between intragenic and extragenic suppressors. Meanwhile, the suppressor mutants were backcrossed to *cop1-6*. The phenotype of F1 and the segregation ratio in the F2 generations in the dark were analyzed to identify whether the suppression phenotype is caused by a monogenic recessive mutation.

### Map-based cloning of *csu2-4*

Rough mapping was performed as described [41]. We crossed *csu2-4 cop1-6* (Col background) with Landsberg containing a *cop1-6* mutation to generate the mapping population. F2 generation seeds were sown on plates containing 1×MS medium, and grown in darkness at 22°C for five days. The suppressor seedlings with long hypocotyl and apical hook were then picked for Genomic DNA extraction and mapping. The markers used for mapping were designed based on the *Arabidopsis* Mapping Platform (http://amp.genomics.org.cn) and the standards described previously [56]. *CSU2* was rough mapped to a ~250 kb region between markers 1-U89959-0145 and 1-AC022521-0169 on the left arm of chromosome 1.

### SOLiD sequencing and mutation identification

SOLiD sequencing and mutation identification was performed as previously described [41]. The fragment libraries were created using the SOLiD Fragment library construction procedures according to the manufacturer’s instructions (Life Technologies, Carlsberg, USA). The libraries were sequenced on a SOLiD5500 sequencer according to the manufacturer’s instructions (Life Technologies, Carlsberg, USA). Mapping of sequencing reads to the *Arabidopsis thaliana* reference genome (TAIR10) and single nucleotide polymorphism (SNP) calling were accomplished using LifeScope v2.5. SNPs were then sorted into four categories (EMS induced homozygous, EMS induced heterozygous, other homozygous and other heterozygous). Candidate homozygous EMS induced SNPs were identified in windows with reduced heterozygosity in the regions identified by physical mapping using in house scripts.

### Measurement of hypocotyl and root length

To measure the hypocotyl and root length of seedlings, seeds were sown on horizontal or vertical plates and stratified at 4°C in darkness for three days, and then kept in continuous white light for eight h in order to induce uniform germination. The seeds were then transferred to dark, white, blue, red, and far-red light conditions, and grown at 22°C for five days [41]. The hypocotyl and root length of seedlings was measured using ImageJ software.

### Construction of plasmids

The full-length CSU2 open reading frame (ORF), CSU2 coiled-coil domain fragment and CSU2 lacking coiled-coil domain fragment were cloned into the pDONR-221 vector (Invitrogen) and introduced into the plant binary vector pEarley Gateway 103, pEarley Gateway 104 or pEarley Gateway 203 [57] under the 35S promoter using Gateway LR Clonase enzyme mix (Invitrogen). pEarley Gateway-CSU2-GFP, pEarley Gateway-YFP-CSU2, pEarley Gateway-Myc-CSU2, pEarley Gateway-CSU2 coil-GFP, and pEarleyGateway-CSU2Δcoil-GFP constructs were generated.

pB42AD-COP1, pB42AD-COP1N282, pB42AD-COP1ΔRing, pB42AD-COP1 Ring, pB42AD-COP1 coil, and pB42AD-COP1 WD40 constructs were described previously [17].To generate pLexA-CSU2, pLexA-CSU2 coil and pLexA-CSU2 Δcoil constructs, full-length CSU2, CSU2 coiled-coil domain and CSU2 lacking coiled-coil domain fragment were amplified by PCR with the respective pairs of primers and then cloned into the *EcoR*I/XhoI sites of the pLexA vector (BD Clontech). To produce the constructs for BiFC assays, each full-length CSU2 or COP1 fragments was amplified by PCR with the respective pairs of primers and then cloned into the *NcoI*/*NotI* sites of pSY728 or pSY738 vector [58], respectively. COP1-Flag construct was prepared with modified versions of pCombia1300 plasmid. pJIM-35S-HA-HY5 [59], pCombia1300-35S-GFP [60], and pCombia1300-35S-P19 [61] constructs were described previously. To produce pCold-TF-COP1, full-length COP1 were amplified by PCR and then cloned into the KpnI/PstI sites of the pCold-TF vector (Takara). To generate pET28a-CSU2 and pET28a-CSU2 Δcoil, full-length CSU2 or CSU2 Δcoil fragment lacking CSU2 coiled-coil domain were amplified by PCR and then cloned into the *NdeI/XhoI* sites of the pET28a vector, respectively. The primers used for plasmids construction were listed in S1 Table.

### Yeast-two hybrid assays

The LexA-based yeast two-hybrid system (BD Clontech) was used for the assays. The respective combinations of LexA and AD fusion plasmids were co-transformed into the yeast strain EGY48. Yeast transformation and the β-galactosidase activity assays were performed as described in the Yeast Protocols Handbook (BD Clontech).

### Bimolecular Fluorescence Complementation assays (BiFC)

Each pair of recombinant constructs encoding nYFP and cYFP fusions was co-bombarded into onion epidermal cells and incubated in 1×MS solid media containing 4% sucrose for 24 h at 22°C in darkness, followed by observation and image analysis by using confocal microscopy.

### Fluorescence Resonance Energy Transfer (FRET) and co-localization assays

FRET and co-localization assay experiments were performed according to the standards outlined in previous research [19]. For FRET assays, the pAM-PAT-35SS-YFP-COP1 [41], pAM-PAT-35SS-CFP-CSU2 (this study), overexpression constructs were introduced into onion epidermal cells by particle bombardment and incubated, and live cell images were acquired using an Axiovert 200 microscope equipped with a laser scanning confocal imaging LSM 510 META system (Carl Zeiss). Cells were visualized at 24 h after particle bombardment using the confocal microscope. The multitracking mode was used to eliminate spillover between fluorescence channels. The CFP was excited by a laser diode 405 laser and the YFP by an argon-ion laser, both at low intensities. Regions of interest were selected and bleached with 100 iterations using the argon-ion laser at 100%. For co-localization assays, respective combination of pRTL2-35S-COP1 [19], pEarly Gateway-35S-CSU2-GFP (this study), pEarly Gateway-35S-CSU2 coil-GFP (this study), and pEarly Gateway-35S-CSU2Δcoil-GFP (this study) constructs were introduced into onion epidermal cells by particle bombardment, and incubated in darkness for 24 h. The cells were then analyzed by confocal microscopy.

### Semi-quantitative RT-PCR and quantitative Real-Time PCR

Total RNA was extracted from five-d-old *Arabidopsis* seedlings grown under white light using the RNeasy plant mini kit (QIAGEN). cDNAs were synthesized from 2 mg of total RNA using the SuperScript II first-strand cDNA synthesis system (Fermentas) according to the manufacturer’s instructions. Then, cDNA were subjected to PCR or real-time qPCR assays. Quantitative real-time qPCR was performed using the CFX96 real-time PCR detection system (Applied Biosystems) and SYBR Green PCR Master Mix (Takara). PCR was performed in triplicate for each sample, and the expression levels were normalized to that of a *PP2A* gene.

### *In vitro* ubiquitination assays

*In vitro* ubiquitination assays were performed as previously described [41], with some minor modifications. Ubiquitination reaction mixtures (60 μL) contained 30 ng of UBE1 (E1; Boston Biochem), UbcH5b (E2; Boston Biochem), and 500 ng of HA-tagged ubiquitin (HA-Ub; Boston Biochem) in a reaction buffer containing 50 mM Tris at pH 7.5, 10 mM MgCl_2_, 2 mM ATP, and 0.5 mM DTT. 500 ng 6×His-TF, 500 ng 6×His-TF-COP1 (previously incubated with 20 μM zinc acetate), 500 ng 6×His-CSU2, and 500 ng 6×His-CSU2 Δcoil were applied in the reactions as indicated. After 2 h incubation at 30°C, the reactions were stopped by adding 5×sample buffer. One-half of each mixture (30 μL) was then separated onto 8% SDS-PAGE gels. Ubiquitinated TF-COP1 was detected using anti-ubiquitin (Santa Cruz), and anti-HA (Sigma-Aldrich) antibodies, respectively.

### *In vitro* protein degradation assays

*In vitro* protein degradation assays were performed as described [62] with minor modification. For *in vitro* protein degradation analysis, *Agrobacterium tumefaciens* strains carrying constructs of p19 (for suppressing PTGS) together with HA-HY5, COP1-Flag, myc-CSU2, myc-CSU2Δcoil, or GFP (internal control) plasmids were co-infiltrated in *Nicotiana benthamiana* leaves, separately. One day after infiltration, a HA-HY5 sample was harvested. COP1-Flag sample, myc-CSU2 sample and GFP sample were collected after three days infiltration, individually. These four samples were separately extracted in native extraction buffer (50 mM Tris-MES pH 8.0, 0.5 M sucrose, 1 mM MgCl_**2**_, 10 mM EDTA, 5 mM DTT, 10 mM PMSF, 1×protease inhibitor cocktail (Roche)). Then, 100 μg HA-HY5 extract was mixed with 100 μg Flag-COP1, 100 μg GFP, 100 μg or 200 μg myc-CSU2 and myc-CSU2 Δcoil extract as indicated. A final concentration of 10 μM ATP was added to the reaction samples to preserve the function of the ubiquitination and 26S proteasome. For the proteasome inhibition, a final concentration of 50 μM MG132 was added to the corresponding mixtures. The mixtures were incubated at 4°C with gentle shaking for 6 h. Reaction was stopped by the addition of 5×SDS sample buffer and boiling for 10 min before protein gel analysis. The primary antibodies used in this study were anti-Flag (Sigma-Aldrich), anti-HA (Sigma-Aldrich), anti-GFP (BD Clontech), and anti-myc (Sigma-Aldrich).

### Statistical analysis

Statistical analysis was performed by using GraphPad Prism 6 (GraphPad Software). To determine statistical significance, we employed one-way ANOVA with Tukey’s posthoc test. The difference was considered significant at P < 0.05.

### Accession numbers

Sequence data from this article can be found in the *Arabidopsis* Genome Initiative database under the following accession numbers: *CSU2* (At1g02330), *COP1* (AT2G32950), *HY5* (AT5G11260).

## Supporting Information

S1 Fig *csu2* has little effect on the splicing pattern of *COP1-6* mRNA. PCR products were generated from Col, *cop1-6* and *csu2 cop1-6* mutant seedlings using primers corresponding to the adjacent exons, and were separated on a 12% acrylamide gel followed by silver staining. M, molecular size markers in base pairs.
(TIF)

S2 Fig Alignment of CSU2 with its orthologs from other species. *Oryza sativa* (NP_001049735), *Drosophila melanogaster* (NP_573288), *Danio rerio* (NP_001007435), *Mus musculus* (NP_659134) and *Homo sapiens* (NP_057604). Black boxes are identical residues; dots indicate gaps. The putative coiled-coil domains are underlined in red.
(TIF)

S3 Fig Mutations in *CSU2* partially suppress *cop1-6* in the light. Hypocotyl phenotype and length (millimeter) of five-d-old Col, *cop1-6* and *csu2 cop1-6* mutant seedlings grown under white light (33.3 μmol/m^2^/s) (A-B); blue light (0.62 μmol/m^2^/s) (C-D); far-red light (1.46 μmol/m^2^/s) (E-F); and red light (6.78 μmol/m^2^/s) (G-H). Data are means ± SE; n≥20. Letters above the bars indicate significant differences as determined by one-way ANOVA with Tukey’s posthoc analysis (P<0.05). The experiment was repeated three times with similar results.
(TIF)

S4 Fig Mutations in *CSU2* partially suppress the adult dwarf phenotype of *cop1-6*. Morphology of Col, *cop1-6* and *csu2 cop1-6* mutants were grown in soil under long-day conditions for 30 days.
(TIF)

S5 Fig The *csu2* mutants are hyposensitive to white light. (A) Hypocotyl length of Col and *csu2* mutant seedlings grown in various fluence rates of white light for fivedays. Data are means ± SE; n≥20. Letters above the bars indicate significant differences as determined by one-way ANOVA with Tukey’s posthoc analysis (P<0.05). The experiment was repeated three times with similar results.
(TIF)

S6 Fig Hypocotyl phenotype and length of *csu2* mutant seedlings grown in the light or dark. Hypocotyl phenotype and length of five-d-old Col and *cus2* mutant seedlings grown under blue light (A), red light (B), and far-red (C) conditions. Data are means ± SE; n≥20. The experiments were performed 3 times with similar results. The graphs depict one of these experiments.
(TIF)

S7 Fig FRET analysis between YFP-COP1 and CSU2-CFP analyzed by acceptor bleaching in nuclei. The top panels in (A) show representative pre-bleach nuclei co-expressing YFP-COP1 and CSU2-CFP excited with a 514- or a 405-nm laser, resulting in emission from YFP (red) or CFP (green), respectively. The region of interest in the nucleus (dotted) was bleached with the 514- nm laser. The bottom panels in (A) show the same nuclei after bleaching excited with a 514- or 405-nm laser. The relative intensities of both YFP and CFP inside the nucleus were measured once before and twice after the bleaching, as indicated in (B). An increase in donor fluorescence (green) is seen only if a protein–protein interaction occurs. (C-D) Absence of FRET between unfused YFP and CFP-CSU2.
(TIF)

S8 Fig CSU2-GFP is localized to nucleus both in the dark and light. Analysis of CSU2-GFP localization with fluorescence microscopy. *CSU2-GFP csu2-2* transgenic seedlings were grown in the dark and white light for five days. The pictures represent images taken from hypocotyls or roots. GFP, GFP channel image; Dic, differential interference contrast in light microscope mode; Merge, merged images of GFP and Dic. Bar = 50μm.
(TIF)

S9 Fig COP1 does not regulate CSU2 protein level *in vivo*. (A-D) Analysis of YFP-CSU2 in hypocotyl (A) or root (C) with fluorescence microscopy. *YFP*-*CSU2 csu2-2* and *YFP*-*CSU2 csu2-2 cop1-6* transgenic seedlings were grown in the dark and various light conditions for five days. The pictures represent images taken from hypocotyls or roots. GFP, GFP channel image; Dic, differential interference contrast in light microscope mode; Merge, merged images of GFP and Dic. Bar = 50μm. Relative YFP fluorescence intensity in hypocotyl (B) or root (D) of *YFP-CSU2 csu2-2* and *YFP-CSU2 csu2-2 cop1-6* transgenic seedlings were grown in the dark and various light conditions for 5 days. Data were obtained from three independent experiments. At least 10 seedlings were measured each time. Fluorescence intensity was measured using Image J software.
(TIF)

S10 Fig Immunoblot analysis of YFP-CSU2 protein levels in *YFP*-*CSU2 csu2-2* and *YFP*-*CSU2 csu2-2 cop1-6* transgenic seedlings. *YFP-CSU2 csu2-2* and *YFP-CSU2 csu2-2 cop1-6* transgenic seedlings were grown in various light conditions (dark, white, blue, red or far-red) for five days. *csu2-2* mutant samples were used as negative control.
(TIF)

S11 Fig Loss of CSU2 has no effect on phyA, phyB, COP1, HY5 and SPA1-4 protein levels *in vivo*. (A) Protein levels of COP1, HY5, SPA1-4, phyA and phyB in Col, and *csu2* seedlings grown in darkness for five days as detected by COP1, HY5, SPA1-4, phyA and phyB antibodies, respectively. *cop1-6*, *spa123*, *spa124*,and *phyab* mutant samples were used as negative control, respectively. (B) Protein levels of COP1, HY5, phyA and phyB in Col and *csu2* seedlings grown in white light for five days as detected by COP1, phyA and phyB antibodies, respectively. *cop1-6* and *phyab* mutant samples were used as negative control, respectively.
(TIF)

S12 Fig myc-CSU2, CSU2-GFP and YFP-CSU2 complement the short root phenotype of *csu2-2*. (A) Semi-quantitative RT and (B) quantitative real-time PCR showing *CSU2* gene expression in the Col, *myc*-*CSU2 csu2-2*, *CSU2-GFP csu2-2* and *YFP-CSU2 csu2-2* transgenic seedlings grown in white light for five days. (C) Root phenotype of Col, *myc*-*CSU2 csu2-2*, *CSU2-GFP csu2-2* and *YFP-CSU2 csu2-2* transgenic seedlings grown in constant white light for 5 days.
(TIF)

S13 Fig *csu2* does not suppress *cop1-1*, *cop1-4* and *det1-1* in darkness. (A-B) Hypocotyl phenotype and length of Col, *csu2*, *cop1-1*, *cop1-4*, *det1-1*, *cus2 cop1-1*, *csu2 cop1-4*, and *csu2 det1-1* mutant seedlings grown in darkness for five days. Data are means ± SD; n≥20. (C) Protein gel blot analysis of the COP1 protein in Col, *cop1-1*, *cop1-4* and *cop1-6* mutant seedlings. Col, *cop1-1*, *cop1-4* and *cop1-6* mutant seedlings were grown in the dark or white light for five days.
(TIF)

S1 Table List of primers used in this study. (DOC)

## References

[1] Sullivan JA, Deng XW. From seed to seed: the role of photoreceptors in *Arabidop sis* development. Dev Biol. 2003; 260: 289–297. 12921732 10.1016/s0012-1606(03)00212-4

[2] Bae G, Choi G. Decoding of light signals by plant phytochromes and their interacting proteins. Annu Rev Plant Biol. 2008; 59: 281–311. 10.1146/annurev.arplant.59.032607.092859 18257712

[3] Chen M, Chory J. Phytochrome signaling mechanisms and the control of plant development. Trends Cell Biol. 2011; 21: 664–671. 10.1016/j.tcb.2011.07.002 21852137 PMC3205231

[4] Ahmad M, Cashmore AR. Seeing blue the discovery of cryptochrome. Plant Mol Biol. 1996; 30: 851–861. 8639745 10.1007/BF00020798

[5] Christie JM. Phototropin blue-light receptors. Annu Rev Plant Biol. 2007; 58: 21–45. 17067285 10.1146/annurev.arplant.58.032806.103951

[6] Rizzini L, Favory JJ, Cloix C, Faggionato D, O'Hara A, Kaiserli E, et al Perception of UV-B by the *Arabidopsis* UVR8 protein. Science. 2011; 332: 103–106. 10.1126/science.1200660 21454788

[7] Tepperman JM, Zhu T, Chang HS, Wang X, Quail PH. Multiple transcription-factor genes are early targets of phytochrome A signaling. Proc Natl Acad Sci USA. 2001; 98: 9437–9442. 11481498 10.1073/pnas.161300998 PMC55439

[8] Tepperman JM, Hudson ME, Khanna R, Zhu T, Chang SH, Wang X, et al Expression profiling of phyB mutant demonstrates substantial contribution of other phytochromes to red light-regulated gene expression during seedling de-etiolation. Plant J. 2004; 38: 725–739. 15144375 10.1111/j.1365-313X.2004.02084.x

[9] Chen F, Li B, Li G, Charron JB, Dai M, Shi X, et al *Arabidopsis* Phytochrome A directly targets numerous promoters for individualized modulation of genes in a wide range of pathways. Plant Cell. 2014; 26: 1949–1966. 24794133 10.1105/tpc.114.123950 PMC4079361

[10] Wei N, Deng XW. The role of the COP/DET/FUS genes in light control of *Arabidopsis* seedling development. Plant Physiol. 1996; 112: 871–878. 8938399 10.1104/pp.112.3.871 PMC158013

[11] Hoecker U, Quail PH. The phytochrome A-specific signaling intermediate SPA1 interacts directly with COP1, a constitutive repressor of light signaling in *Arabidopsis* . J Biol Chem. 2001; 276: 38173–38178. 11461903 10.1074/jbc.M103140200

[12] Zhu D, Maier A, Lee JH, Laubinger S, Saijo Y, Wang H, et al Biochemical characterization of *Arabidopsis* complexes containing CONSTITUTIVELY PHOTOMORPHOGENIC1 and SUPPRESSOR OF PHYA proteins in light control of plant development. Plant Cell. 2008; 20: 2307–2323. 10.1105/tpc.107.056580 18812498 PMC2570740

[13] Torii KU, McNellis TW, Deng XW. Functional dissection of *Arabidopsis* COP1 reveals specific roles of its three structural modules in light control of seedling development. EMBO J. 1998; 17: 5577–5587. 9755158 10.1093/emboj/17.19.5577 PMC1170886

[14] Seo HS, Yang JY, Ishikawa M, Bolle C, Ballesteros ML, Chua NH. LAF1 ubiquitination by COP1 controls photomorphogenesis and is stimulated by SPA1. Nature 2003; 423: 995–999. 12827204 10.1038/nature01696

[15] Osterlund MT, Hardtke CS, Wei N, Deng XW. Targeted destabilization of HY5 during light-regulated development of *Arabidopsis* . Nature. 2000; 405: 462–466. 10839542 10.1038/35013076

[16] Holm M, Ma LG, Qu LJ, Deng XW. Two interacting bZIP proteins are direct targets of COP1-mediated control of light-dependent gene expression in *Arabidopsis* . Genes Dev. 2002 16; 1247–1259. 12023303 10.1101/gad.969702 PMC186273

[17] Saijo Y, Sullivan JA, Wang H, Yang J, Shen Y, Rubio V, et al The COP1-SPA1 interaction defines a critical step in phytochrome A-mediated regulation of HY5 activity. Gene Dev. 2003; 17: 2642–2647. 14597662 10.1101/gad.1122903 PMC280614

[18] Jang IC, Yang JY, Seo HS, Chua NH. HFR1 is targeted by COP1 E3 ligase for post-translational proteolysis during phytochrome A signaling. Genes Dev. 2005; 19: 593–602. 15741320 10.1101/gad.1247205 PMC551579

[19] Datta S, Johansson H, Hettiarachchi C, Irigoyen ML, Desai M, Rubio V, et al LZF1/SALT TOLERANCE HOMOLOG3, an *Arabidopsis* B-box protein involved in light-dependent development and gene expression, undergoes COP1-mediated ubiquitination. Plant Cell. 2008; 20: 2324–2338. 10.1105/tpc.108.061747 18796637 PMC2570732

[20] Luo Q, Lian HL, He SB, Li L, Jia KP, Yang HQ. COP1 and phyB physically interact with PIL1 to regulate its stability and photomorphogenic development in *Arabidopsis* . Plant Cell. 2014; 26: 2441–2456. 24951480 10.1105/tpc.113.121657 PMC4114944

[21] Jang S, Marchal V, Panigrahi KC, Wenkel S, Soppe W, Deng XW, et al *Arabidopsis* COP1 shapes the temporal pattern of CO accumulation conferring a photoperiodic flowering response. EMBO J. 2008; 27: 1277–1288. 10.1038/emboj.2008.68 18388858 PMC2291449

[22] Yu JW, Rubio V, Lee NY, Bai S, Lee SY, Kim SS, et al COP1 and ELF3 control circadian function and photoperiodic flowering by regulating GI stability. Mol Cell. 2008; 32: 617–630. 10.1016/j.molcel.2008.09.026 19061637 PMC2651194

[23] Jeong RD, Chandra-Shekara AC, Barman SR, Navarre D, Klessig DF, Kachroo A, et al Cryptochrome 2 and phototropin 2 regulate resistance protein-mediated viral defense by negatively regulating an E3 ubiquitin ligase. Proc Natl Acad Sci USA. 2010; 107: 13538–13543. 10.1073/pnas.1004529107 20624951 PMC2922132

[24] Luo XM, Lin WH, Zhu S, Zhu JY, Sun Y, Fan XY, et al Integration of light- and brassinosteroid-signaling pathways by a GATA transcription factor in *Arabidopsis* . Dev Cell. 2010; 19: 872–883. 10.1016/j.devcel.2010.10.023 21145502 PMC3022420

[25] Dyachok J, Zhu L, Liao F, He J, Huq E, Blancaflor EB. SCAR mediates light-induced root elongation in *Arabidopsis* through photoreceptors and proteasomes. Plant Cell. 2011; 23: 3610–3626. 10.1105/tpc.111.088823 21972261 PMC3229138

[26] Cho SK, Ben, Chaabane S, Shah P, Poulsen CP, Yang SW. COP1 E3 ligase protects HYL1 to retain microRNA biogenesis. Nature Commun. 2014; 5: 5867. 25532508 10.1038/ncomms6867

[27] Chico JM, Fernández-Barbero G, Chini A, Fernández-Calvo P, Díez-Díaz M, Solano R. Repression of Jasmonate-dependent defenses by dhade involves differential regulation of protein stability of MYC transcription factors and their JAZ repressors in *Arabidopsis* . Plant Cell. 2014; 26: 1967–1980. 24824488 10.1105/tpc.114.125047 PMC4079362

[28] Yi C, Li S, Chen X, Wiemer EA, Wang J, Wei N, et al Major vault protein, in concert with constitutively photomorphogenic 1, negatively regulates c-Jun-mediated activator protein 1 transcription in mammalian cells. Cancer Res. 2005; 65: 5835–5840. 15994960 10.1158/0008-5472.CAN-05-0423

[29] Migliorini D, Bogaerts S, Defever D, Vyas D, Denecker G, et al Cop1 constitutively regulates c-Jun protein stability and functions as a tumor suppressor in mice. J Clin Invest. 2011; 121: 1329–1343. 10.1172/JCI45784 21403399 PMC3070608

[30] Vitari AC, Leong KG, Newton K, Yee C, O'Rourke K, Radaelli E, et al COP1 is a tumour suppressor that causes degradation of ETS transcription factors. Nature. 2011; 474: 403–406. 10.1038/nature10005 21572435

[31] Lu G, Zhang Q, Huang Y, Song J, Tomaino R, Ehrenberger T, et al Phosphorylation of ETS1 by Src family kinases prevents its recognition by the COP1 tumor suppressor. Cancer Cell. 2014; 26: 222–234. 10.1016/j.ccr.2014.06.026 25117710 PMC4169234

[32] Lian HL, He SB, Zhang YC, Zhu DM, Zhang JY, Jia KP, et al Blue-light-dependent interaction of cryptochrome 1 with SPA1 defines a dynamic signaling mechanism. Genes Dev. 2011; 25: 1023–1028. 10.1101/gad.2025111 21511872 PMC3093117

[33] Liu B, Zuo Z, Liu H, Liu X, Lin C. *Arabidopsis* cryptochrome 1 interacts with SPA1 to suppress COP1 activity in response to blue light. Genes Dev. 2011; 25: 1029–1034. 10.1101/gad.2025011 21511871 PMC3093118

[34] Zuo Z, Liu H, Liu B, Liu X, Lin C. Blue light-dependent interaction of CRY2 with SPA1 regulates COP1 activity and floral initiation in *Arabidopsis* . Curr Biol. 2011; 21: 841–847. 10.1016/j.cub.2011.03.048 21514160 PMC3150455

[35] Lu XD, Zhou CM, Xu PB, Luo Q, Lian HL, Yang HQ. Red-light-dependent interaction of phyB with SPA1 promotes COP1–SPA1 dissociation and photomorphogenic development in *Arabidopsis* . Mol Plant. 2015; 8: 467–478. 10.1016/j.molp.2014.11.025 25744387

[36] Sheerin DJ, Menon C, Zur Oven-Krockhaus S, Enderle B, Zhu L, Johnen P, et al Light-activated phytochrome A and B interact with members of the SPA family to promote photomorphogenesis in *Arabidopsis* by reorganizing the COP1/SPA Complex. Plant Cell. 2015; 27: 189–201. 10.1105/tpc.114.134775 25627066 PMC4330587

[37] von Arnim AG, Deng XW. Light inactivation of *Arabidopsis* photomorphogenic repressor COP1 involves a cell-specific regulation of its nucleocytoplasmic partitioning. Cell. 1994; 79: 1035–1045. 8001131 10.1016/0092-8674(94)90034-5

[38] von Arnim AG, Osterlund MT, Kwok SF, Deng XW. Genetic and developmental control of nuclear accumulation of COP1, a repressor of photomorphogenesis in *Arabidopsis* . Plant Physiol. 1997; 114: 779–788. 9232869 10.1104/pp.114.3.779 PMC158364

[39] Pacín M, Legris M, Casal JJ. COP1 re-accumulates in the nucleus under shade. Plant J. 2013; 75: 631–641. 10.1111/tpj.12226 23647163

[40] Pacín M, Legris M, Casal JJ. Rapid decline in nuclear constitutive photomorphogenesis 1 abundance anticipates the stabilization of its target elongated hypocotyl 5 in the light. Plant Physiol. 2014; 164: 1134–1138. 10.1104/pp.113.234245 24434030 PMC3938608

[41] Xu D, Lin F, Jiang Y, Huang X, Li J, Ling J, et al The RING-Finger E3 ubiquitin ligase COP1 SUPPRESSOR1 negatively regulates COP1 abundance in maintaining COP1 homeostasis in dark-grown *Arabidopsis* seedlings. Plant Cell. 2014; 26: 1981–1991. 24838976 10.1105/tpc.114.124024 PMC4079363

[42] Xu X, Paik I, Zhu L, Bu Q, Huang X, Deng XW, et al PHYTOCHROME INTERACTING FACTOR1 enhances the E3 ligase activity of CONSTITUTIVE PHOTOMORPHOGENIC1 to synergistically repress photomorphogenesis in *Arabidopsis* . Plant Cell. 2014; 26: 1992–2006. 24858936 10.1105/tpc.114.125591 PMC4079364

[43] McNellis TW, von Arnim AG, Araki T, Komeda Y, Miséra S, Deng XW. Genetic and molecular analysis of an allelic series of *cop1* mutants suggests functional roles for the multiple protein domains. Plant Cell. 1994; 6: 487–500. 8205001 10.1105/tpc.6.4.487 PMC160452

[44] Ang LH, Deng XW. Regulatory hierarchy of photomorphogenic loci: allele-specific and light-dependent interaction between the HY5 and COP1 loci. Plant Cell. 1994; 6: 613–628. 8038602 10.1105/tpc.6.5.613 PMC160463

[45] Ang LH, Chattopadhyay S, Wei N, Oyama T, Okada K, Batschauer A, et al Molecular interaction between COP1 and HY5 defines a regulatory switch for light control of *Arabidopsis* development. Mol Cell. 1998; 1: 213–222. 9659918 10.1016/s1097-2765(00)80022-2

[46] Selvin PR. The renaissance of fluorescence resonance energy transfer. Nat Struct Biol. 2000; 7: 730–734. 10966639 10.1038/78948

[47] Seo HS, Watanabe E, Tokutomi S, Nagatani A, Chua NH. Photoreceptor ubiquitination by COP1 E3 ligase desensitizes phytochrome A signaling. Genes Dev. 2004; 18: 617–622. 15031264 10.1101/gad.1187804 PMC387237

[48] Chen S, Lory N, Stauber J, Hoecker U. Photoreceptor Specificity in the Light-Induced and COP1-Mediated Rapid Degradation of the Repressor of Photomorphogenesis SPA2 in *Arabidopsis* . PLoS Genet. 2015; 11(9):e1005516 10.1371/journal.pgen.1005516 26368289 PMC4569408

[49] Sassi M, Lu Y, Zhang Y, Wang J, Dhonukshe P, Blilou I, et al COP1 mediates the coordination of root and shoot growth by light through modulation of PIN1- and PIN2-dependent auxin transport in *Arabidopsis* . Development. 2013; 139: 3402–3412. 10.1242/dev.078212 22912415

[50] Lau OS, Deng XW. The photomorphogenic repressors COP1 and DET1: 20 years later. Trends Plant Sci. 2012; 17: 584–593. 10.1016/j.tplants.2012.05.004 22705257

[51] Huang X, Ouyang X, Deng XW. Beyond repression of photomorphogenesis: role switching of COP/DET/FUS in light signaling. Curr Opin Plant Biol. 2014; 21: 96–103. 10.1016/j.pbi.2014.07.003 25061897

[52] Catalá R, Medina J, Salinas J. Integration of low temperature and light signaling during cold acclimation response in *Arabidopsis* . Proc Natl Acad Sci U S A. 2011; 108: 16475–16480. 10.1073/pnas.1107161108 21930922 PMC3182711

[53] Karayekov E, Sellaro R, Legris M, Yanovsky MJ, Casal JJ. Heat shock-induced fluctuations in clock and light signaling enhance phytochrome B-mediated *Arabidopsis* deetiolation. Plant Cell. 2013; 25: 2892–2906. 10.1105/tpc.113.114306 23933882 PMC3784587

[54] Yu Y, Wang J, Zhang Z, Quan R, Zhang H, Deng XW, et al Ethylene promotes hypocotyl growth and HY5 degradation by enhancing the movement of COP1 to the nucleus in the light. PLoS Genet. 2013; 9: e1004025 10.1371/journal.pgen.1004025 24348273 PMC3861121

[55] Stoop-Myer C, Torii KU, McNellis TW, Coleman JE, Deng XW. Short communication: the N-terminal fragment of *Arabidopsis* photomorphogenic repressor COP1 maintains partial function and acts in a concentration-dependent manner. Plant J. 1999; 20: 713–717. 10652143 10.1046/j.1365-313x.1999.00639.x

[56] Jander G, Norris SR, Rounsley SD, Bush DF, Levin IM, Last RL. *Arabidopsis* map-based cloning in the post-genome era. Plant Physiol. 2002; 129: 440–450. 12068090 10.1104/pp.003533 PMC1540230

[57] Earley KW, Haag JR, Pontes O, Opper K, Juehne T, Song K, et al Gateway-compatible vectors for plant functional genomics and proteomics. Plant J. 2006; 45: 616–629. 16441352 10.1111/j.1365-313X.2005.02617.x

[58] Bracha-Drori K, Shichrur K, Katz A, Oliva M, Angelovici R, Yalovsky S, et al Detection of protein-protein interactions in plants using bimolecular fluorescence complementation. Plant J. 2004; 40: 419–427. 15469499 10.1111/j.1365-313X.2004.02206.x

[59] Lee J, He K, Stolc V, Lee H, Figueroa P, Gao Y, et al Analysis of transcription factor HY5 genomic binding sites revealed its hierarchical role in light regulation of development. Plant Cell. 2007; 19: 731–749. 17337630 10.1105/tpc.106.047688 PMC1867377

[60] Wang X, Wu F, Xie Q, Wang H, Wang Y, Yue Y, et al SKIP is a component of the spliceosome linking alternative splicing and the circadian clock in *Arabidopsis* . Plant Cell. 2012; 24: 3278–3295. 22942380 10.1105/tpc.112.100081 PMC3462631

[61] Voinnet O, Rivas S, Mestre P, Baulcombe D. An enhanced transient expression system in plants based on suppression of gene silencing by the p19 protein of tomato bushy stunt virus. Plant J. 2003; 33: 949–956. 12609035 10.1046/j.1365-313x.2003.01676.x

[62] Liu L, Zhang Y, Tang S, Zhao Q, Zhang Z, Zhang H, et al An efficient system to detect protein ubiquitination by agroinfiltration in *Nicotiana benthamiana* . Plant J. 2010; 61: 893–903. 10.1111/j.1365-313X.2009.04109.x 20015064

