# Supplementary figures and images for: Arabidopsis COP1 SUPPRESSOR 2 Represses COP1 E3 Ubiquitin Ligase Activity through Their Coiled-Coil Domains Association
Source: PLoS Genet. 2015 Dec 29;11(12):e1005747. doi: 10.1371/journal.pgen.1005747 (PMC4694719; doi:10.1371/journal.pgen.1005747)

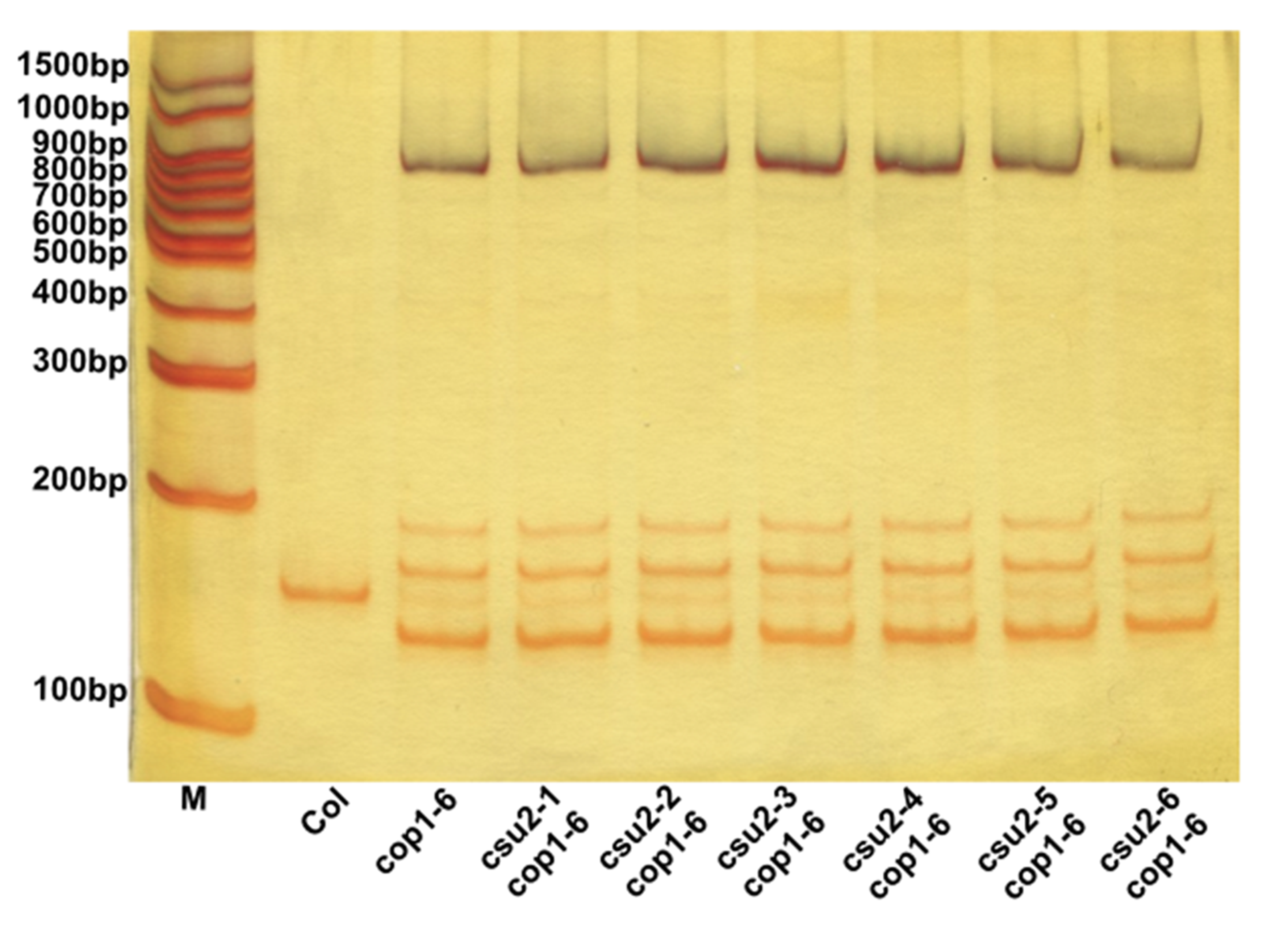

Supplement: S1 Fig — PCR products were generated from Col, cop1-6 and csu2 cop1-6 mutant seedlings using primers corresponding to the adjacent exons, and were separated on a 12% acrylamide gel followed by silver staining. M, molecular size markers in base pairs. (TIF) [file pgen.1005747.s001.tif]

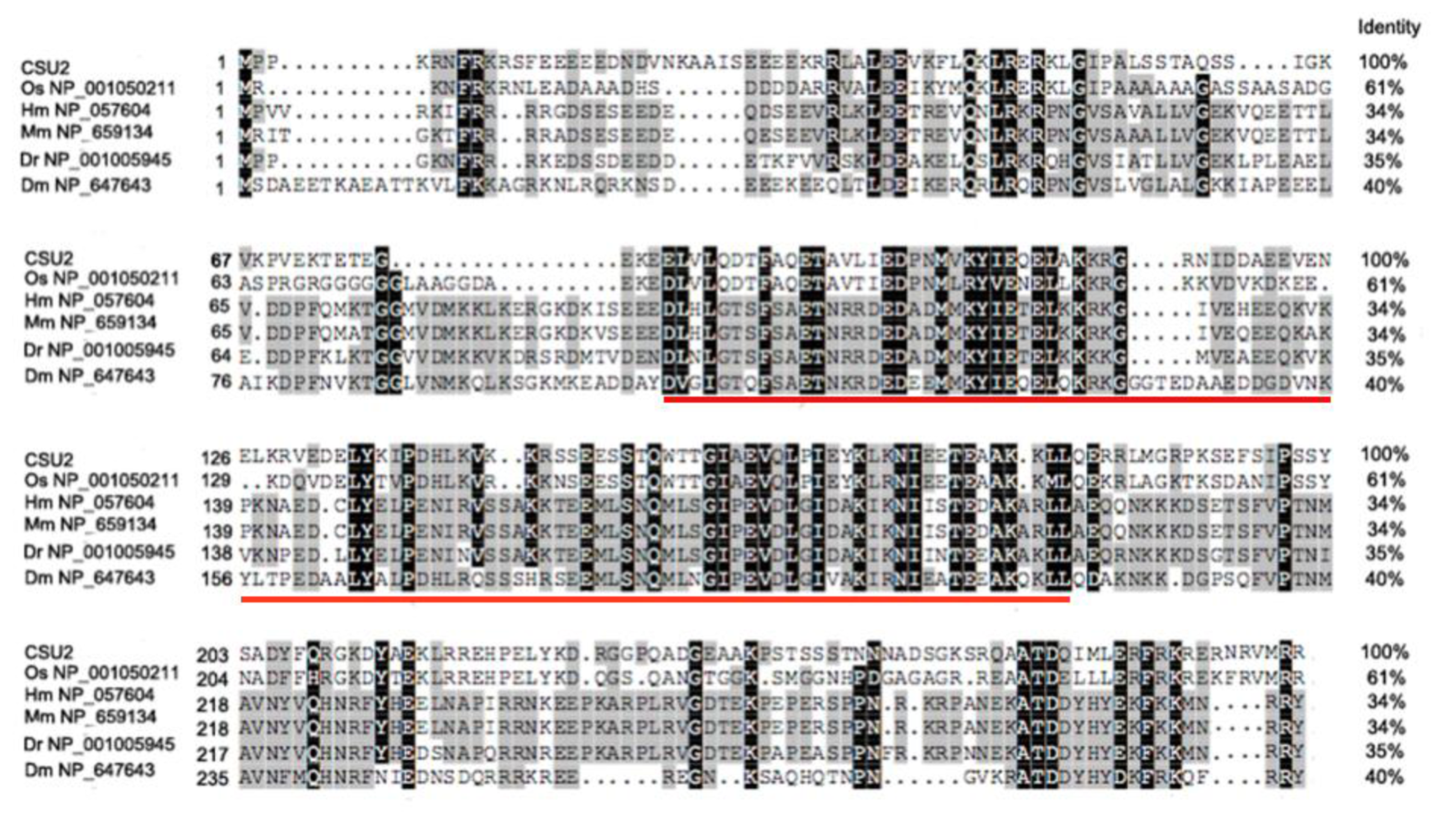

Supplement: S2 Fig — Oryza sativa (NP_001049735), Drosophila melanogaster (NP_573288), Danio rerio (NP_001007435), Mus musculus (NP_659134) and Homo sapiens (NP_057604). Black boxes are identical residues; dots indicate gaps. The putative coiled-coil domains are underlined in red. (TIF) [file pgen.1005747.s002.tif]

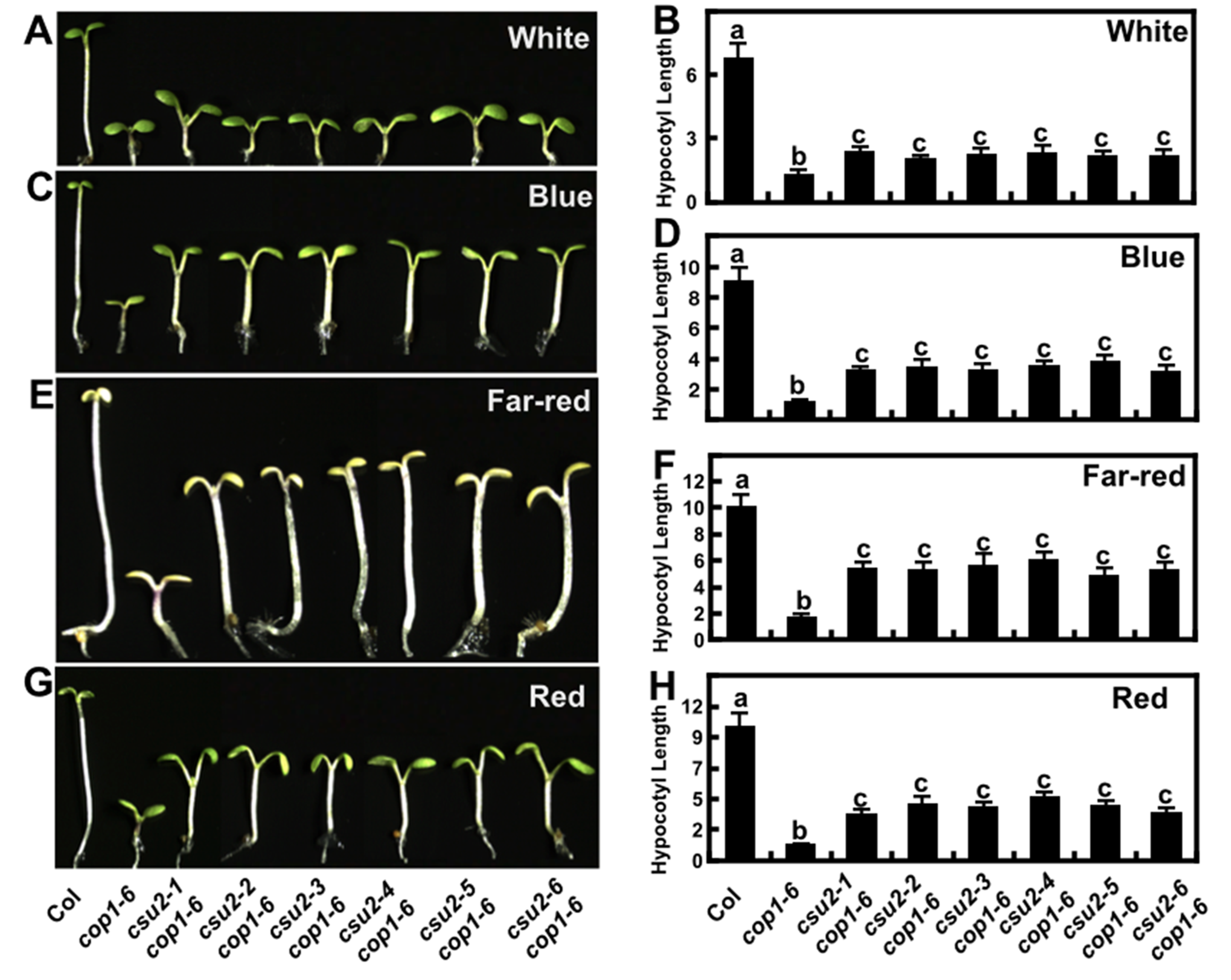

Supplement: S3 Fig — Hypocotyl phenotype and length (millimeter) of five-d-old Col, cop1-6 and csu2 cop1-6 mutant seedlings grown under white light (33.3 μmol/m2/s) (A-B); blue light (0.62 μmol/m2/s) (C-D); far-red light (1.46 μmol/m2/s) (E-F); and red light (6.78 μmol/m2/s) (G-H). Data are means ± SE; n≥20. Letters above the bars indicate significant differences as determined by one-way ANOVA with Tukey’s posthoc analysis (P<0.05). The experiment was repeated three times with similar results. (TIF) [file pgen.1005747.s003.tif]

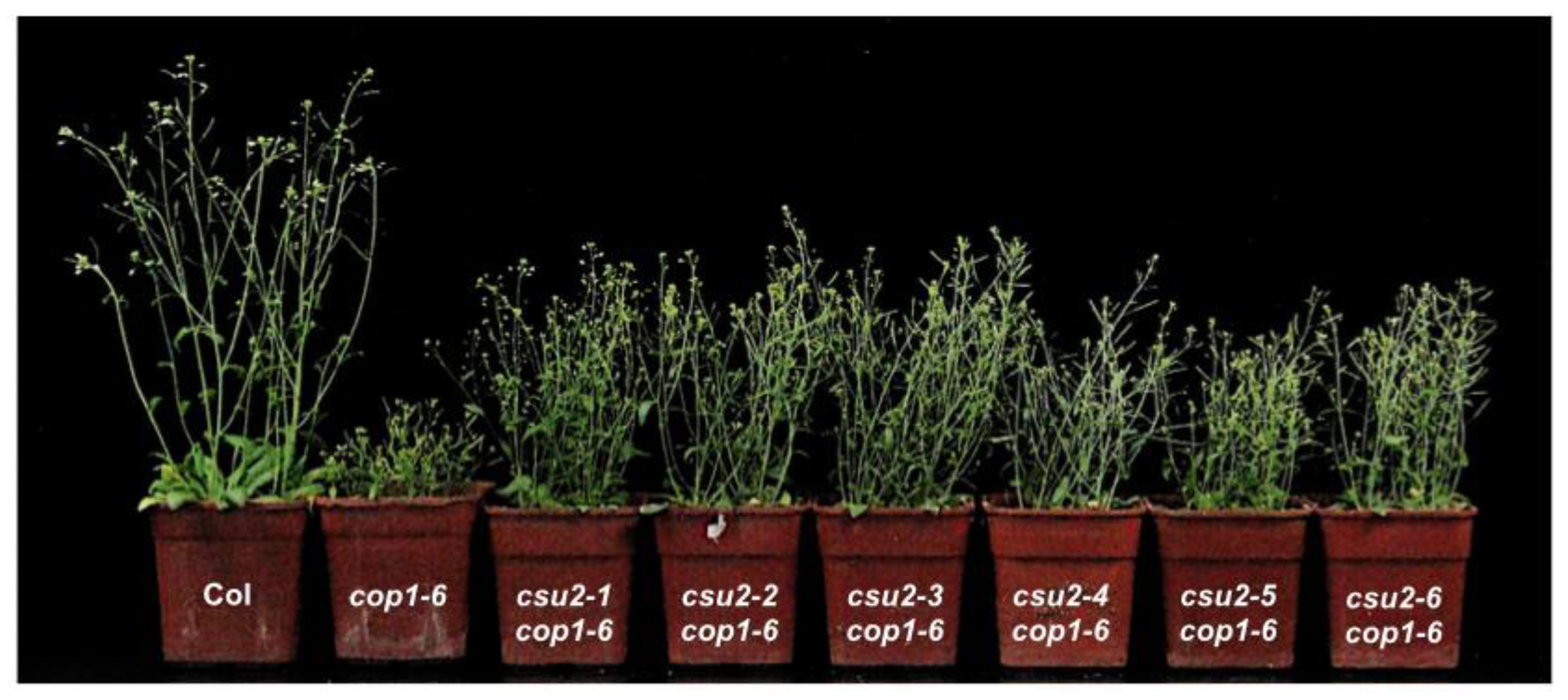

Supplement: S4 Fig — Morphology of Col, cop1-6 and csu2 cop1-6 mutants were grown in soil under long-day conditions for 30 days. (TIF) [file pgen.1005747.s004.tif]

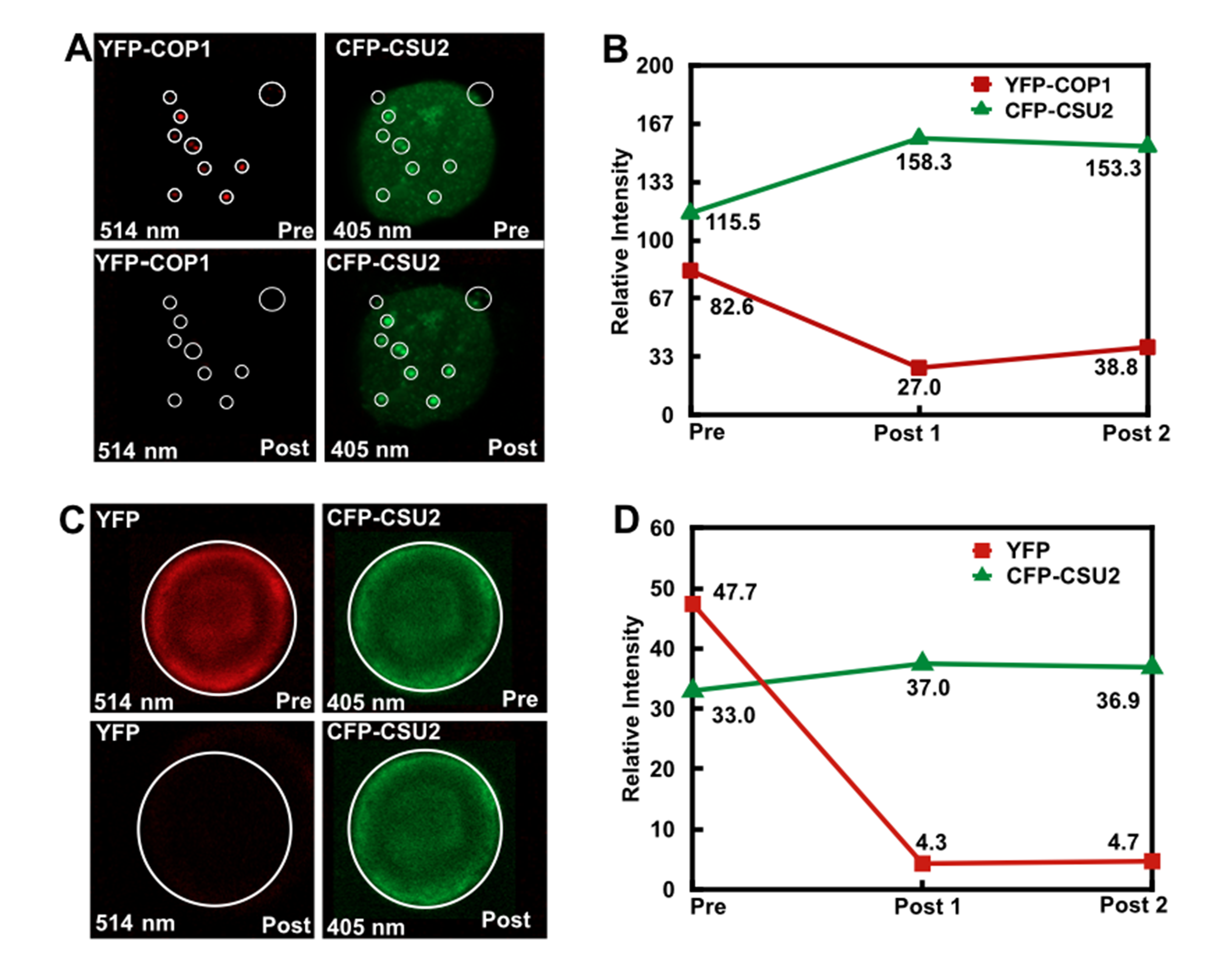

Supplement: S7 Fig — The top panels in (A) show representative pre-bleach nuclei co-expressing YFP-COP1 and CSU2-CFP excited with a 514- or a 405-nm laser, resulting in emission from YFP (red) or CFP (green), respectively. The region of interest in the nucleus (dotted) was bleached with the 514- nm laser. The bottom panels in (A) show the same nuclei after bleaching excited with a 514- or 405-nm laser. The relative intensities of both YFP and CFP inside the nucleus were measured once before and twice after the bleaching, as indicated in (B). An increase in donor fluorescence (green) is seen only if a protein–protein interaction occurs. (C-D) Absence of FRET between unfused YFP and CFP-CSU2. (TIF) [file pgen.1005747.s007.tif]

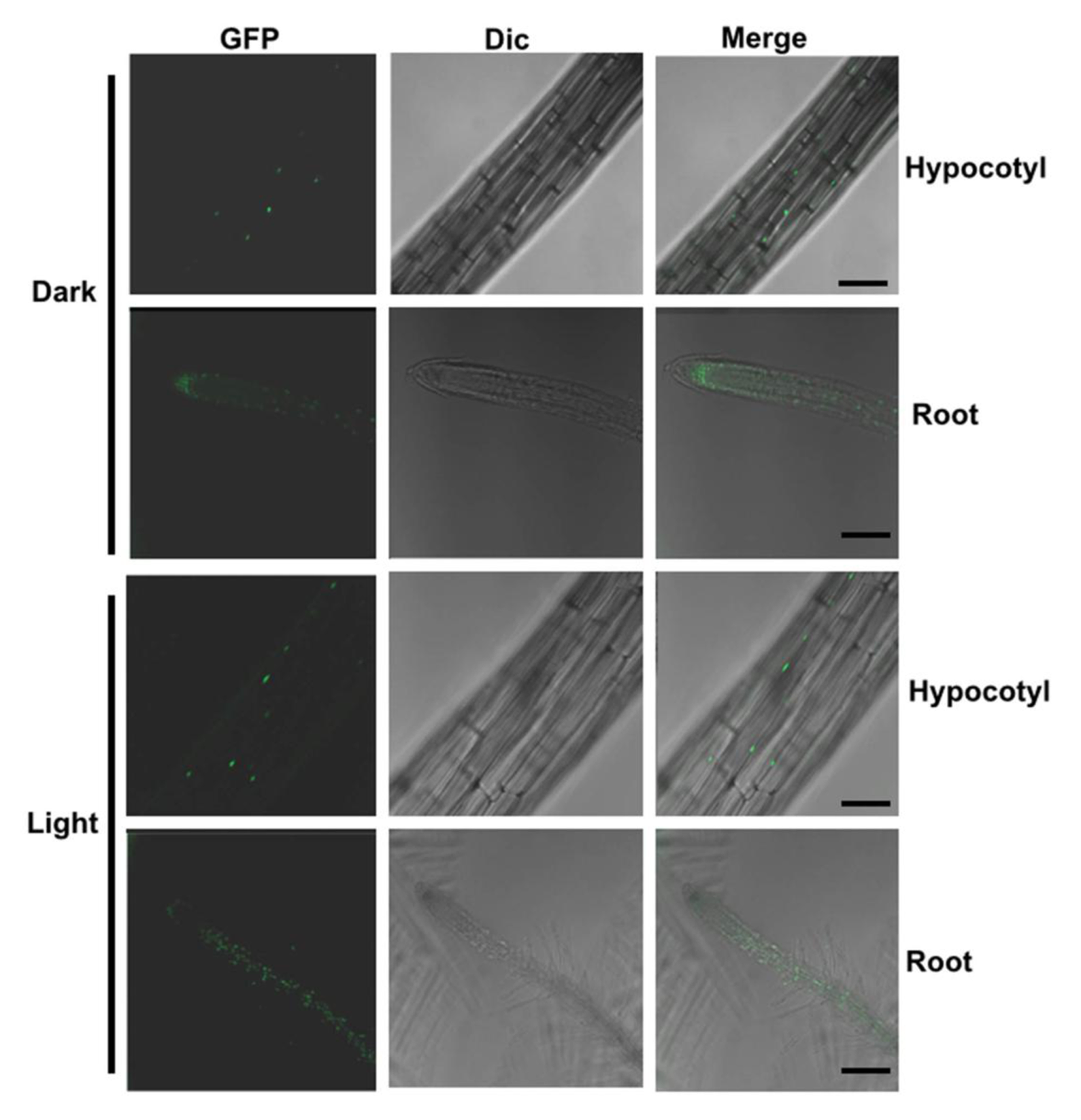

Supplement: S8 Fig — Analysis of CSU2-GFP localization with fluorescence microscopy. CSU2-GFP csu2-2 transgenic seedlings were grown in the dark and white light for five days. The pictures represent images taken from hypocotyls or roots. GFP, GFP channel image; Dic, differential interference contrast in light microscope mode; Merge, merged images of GFP and Dic. Bar = 50μm. (TIF) [file pgen.1005747.s008.tif]

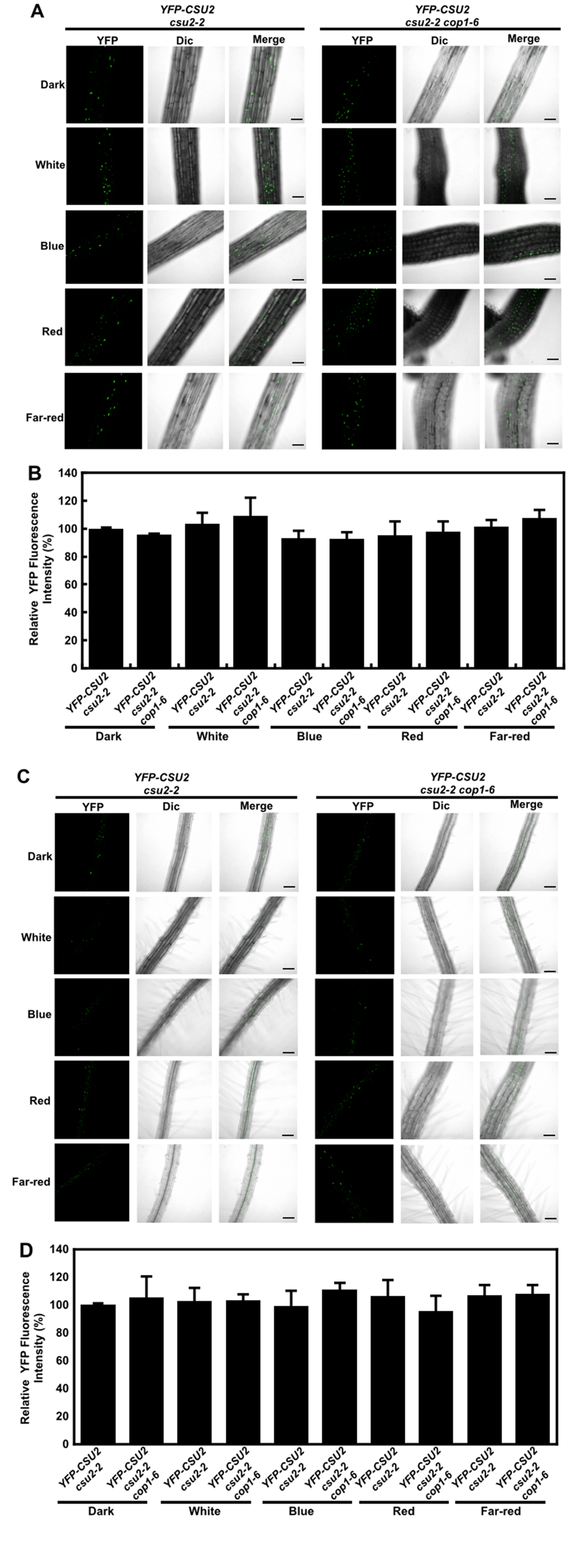

Supplement: S9 Fig — (A-D) Analysis of YFP-CSU2 in hypocotyl (A) or root (C) with fluorescence microscopy. YFP-CSU2 csu2-2 and YFP-CSU2 csu2-2 cop1-6 transgenic seedlings were grown in the dark and various light conditions for five days. The pictures represent images taken from hypocotyls or roots. GFP, GFP channel image; Dic, differential interference contrast in light microscope mode; Merge, merged images of GFP and Dic. Bar = 50μm. Relative YFP fluorescence intensity in hypocotyl (B) or root (D) of YFP-CSU2 csu2-2 and YFP-CSU2 csu2-2 cop1-6 transgenic seedlings were grown in the dark and various light conditions for 5 days. Data were obtained from three independent experiments. At least 10 seedlings were measured each time. Fluorescence intensity was measured using Image J software. (TIF) [file pgen.1005747.s009.tif]

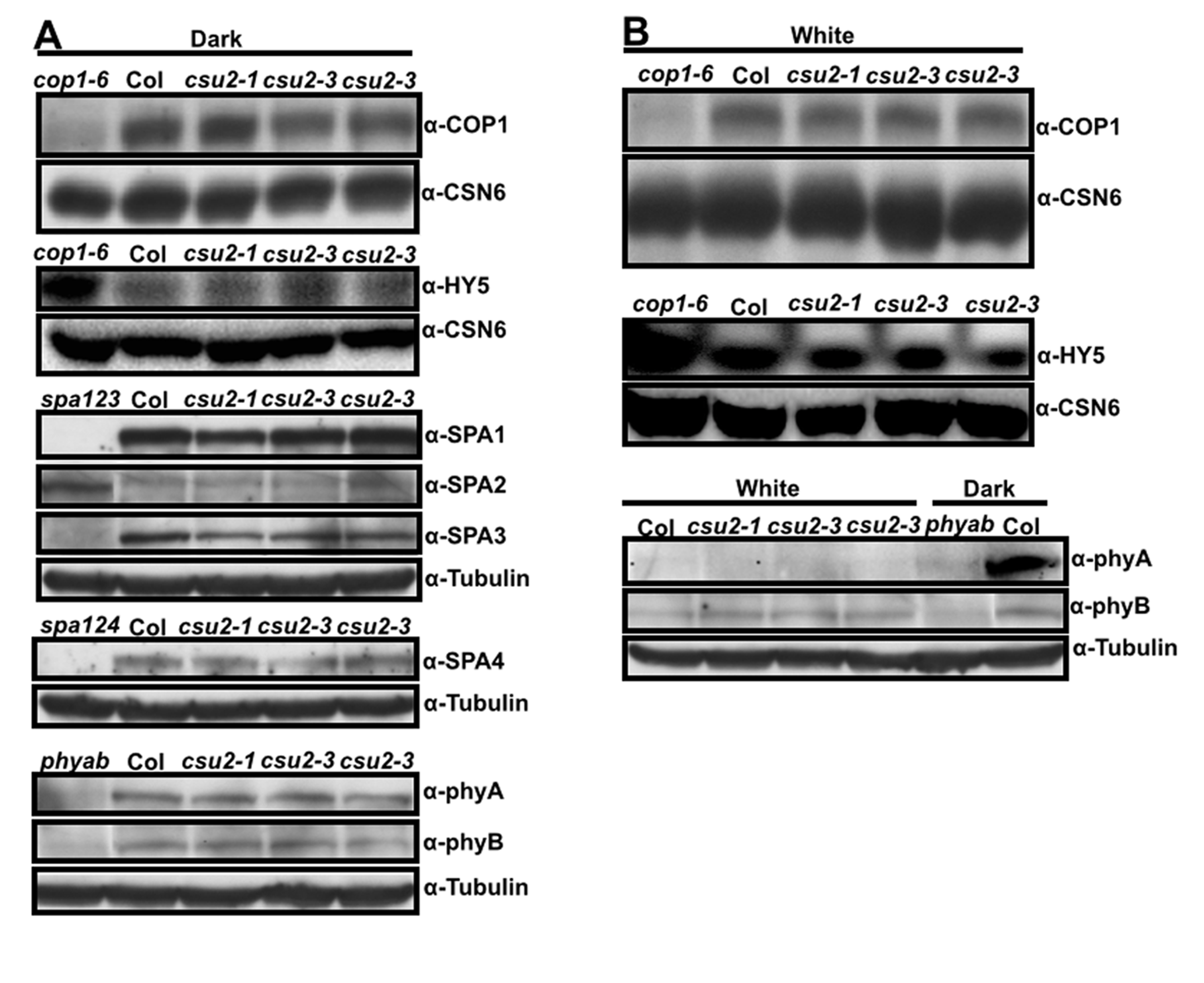

Supplement: S11 Fig — (A) Protein levels of COP1, HY5, SPA1-4, phyA and phyB in Col, and csu2 seedlings grown in darkness for five days as detected by COP1, HY5, SPA1-4, phyA and phyB antibodies, respectively. cop1-6, spa123, spa124,and phyab mutant samples were used as negative control, respectively. (B) Protein levels of COP1, HY5, phyA and phyB in Col and csu2 seedlings grown in white light for five days as detected by COP1, phyA and phyB antibodies, respectively. cop1-6 and phyab mutant samples were used as negative control, respectively. (TIF) [file pgen.1005747.s011.tif]

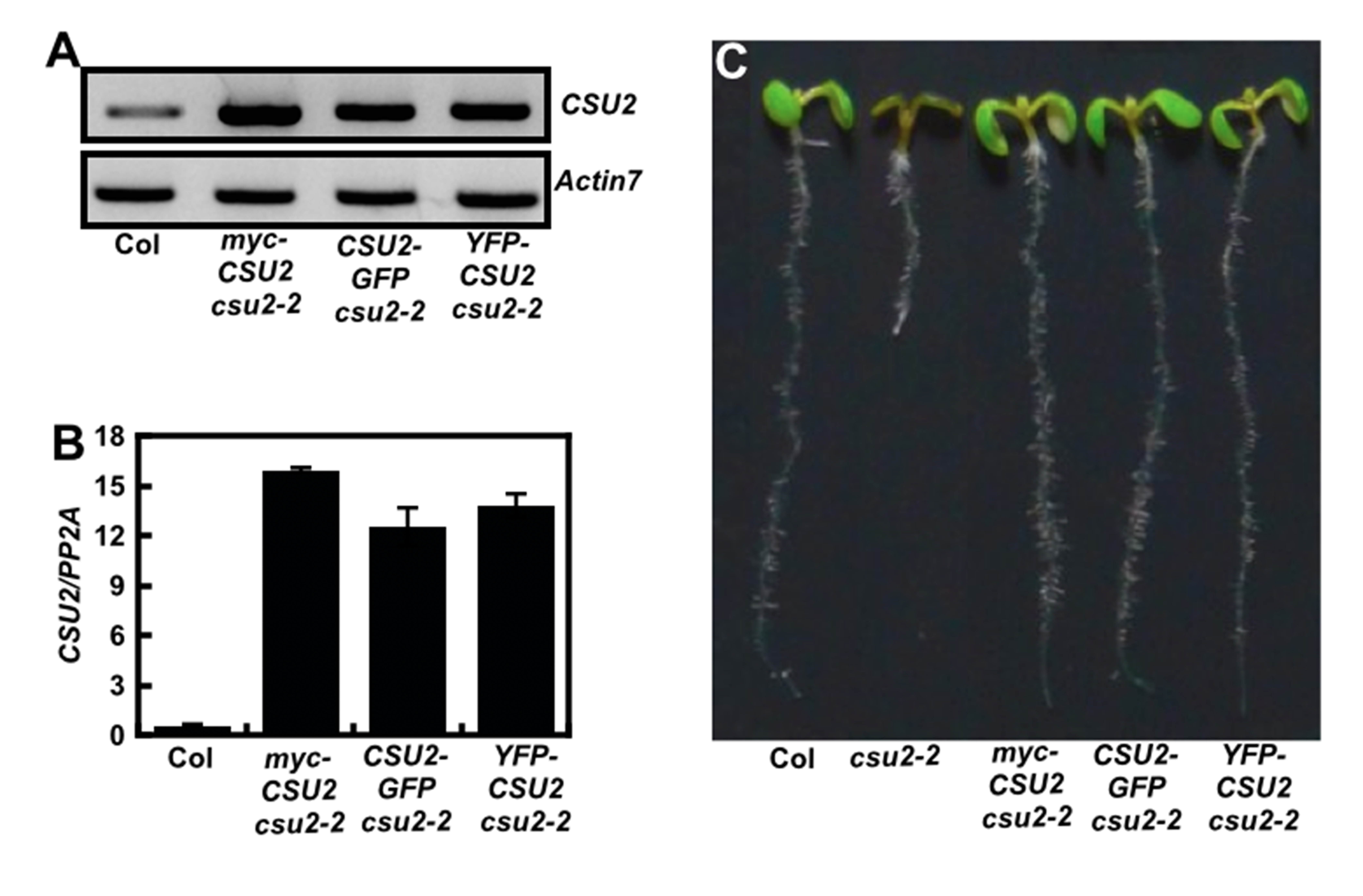

Supplement: S12 Fig — (A) Semi-quantitative RT and (B) quantitative real-time PCR showing CSU2 gene expression in the Col, myc-CSU2 csu2-2, CSU2-GFP csu2-2 and YFP-CSU2 csu2-2 transgenic seedlings grown in white light for five days. (C) Root phenotype of Col, myc-CSU2 csu2-2, CSU2-GFP csu2-2 and YFP-CSU2 csu2-2 transgenic seedlings grown in constant white light for 5 days. (TIF) [file pgen.1005747.s012.tif]

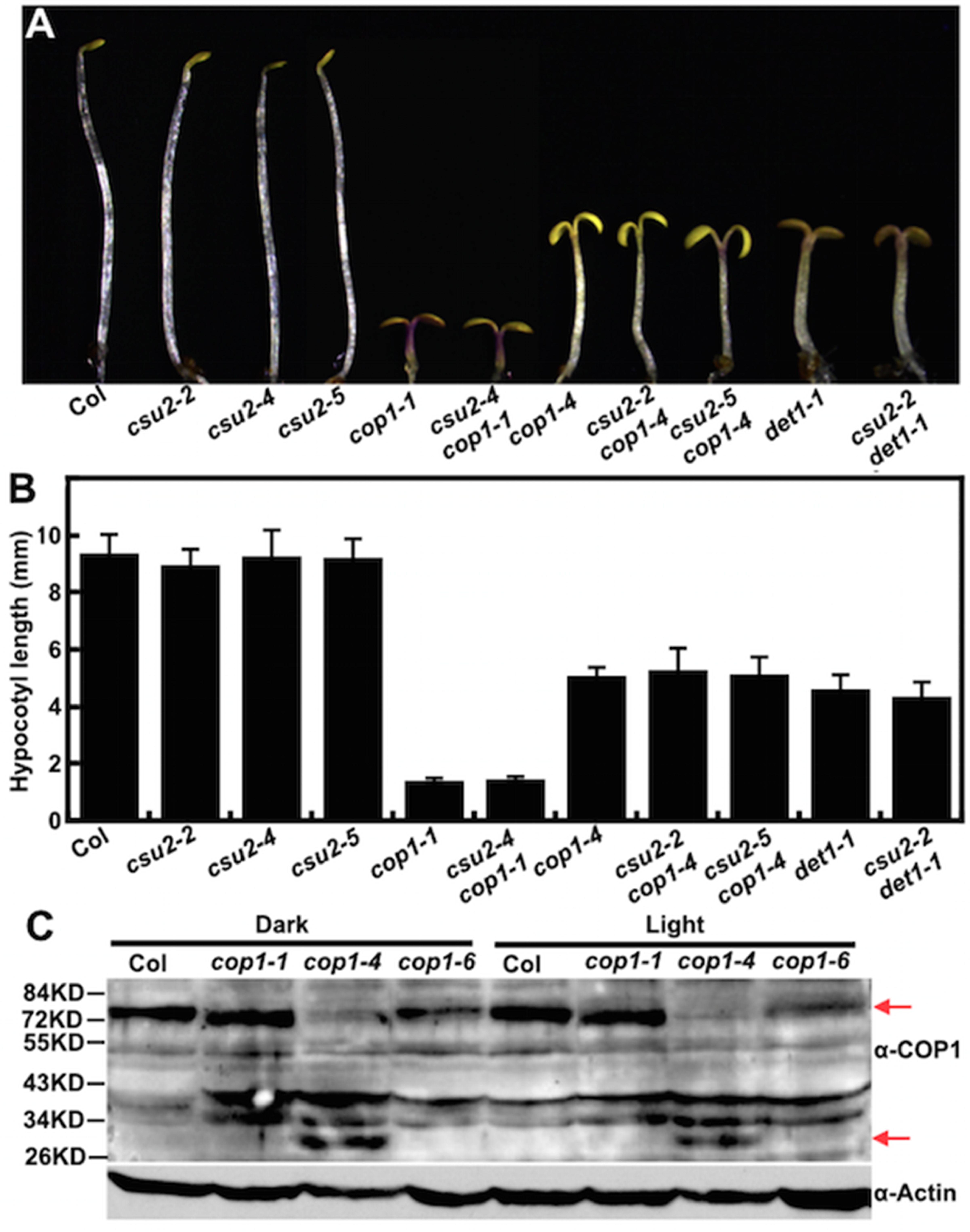

Supplement: S13 Fig — (A-B) Hypocotyl phenotype and length of Col, csu2, cop1-1, cop1-4, det1-1, cus2 cop1-1, csu2 cop1-4, and csu2 det1-1 mutant seedlings grown in darkness for five days. Data are means ± SD; n≥20. (C) Protein gel blot analysis of the COP1 protein in Col, cop1-1, cop1-4 and cop1-6 mutant seedlings. Col, cop1-1, cop1-4 and cop1-6 mutant seedlings were grown in the dark or white light for five days. (TIF) [file pgen.1005747.s013.tif]
