# Supplementary material for: Arabidopsis COP1 SUPPRESSOR 2 Represses COP1 E3 Ubiquitin Ligase Activity through Their Coiled-Coil Domains Association
Source: PLoS Genet. 2015 Dec 29;11(12):e1005747. doi: 10.1371/journal.pgen.1005747 (PMC4694719; doi:10.1371/journal.pgen.1005747)
Supplement: S1 Table — (DOC) [file pgen.1005747.s014.doc]

**S1 Table: List of Primers Used in This Study**

| **Primer name Primer sequences (5’-3’) (Note: The underlined nucleotides indicate the restriction**  **sites for cloning)** | | |
| --- | --- | --- |
| **RT-PCR** | | |
| HY5(F) | CCATCAAGCAGCGAGAGGTC | |
| HY5(R) | GCATTAGAACCACCACCACC | |
| CSU2-RT(F) | TTTTGCTCAAGAGACTGCTG | |
| CSU2-RT(R) | GCTGCTTGTCTGCTTTTCC | |
| Actin1 (F) | CATCAGGAAGGACTTGTACGG | |
| Actin1 (R) | GATGGACCTGACTCGTCATAC | |
| Actin7 (F) | AGGCACCTCTTAACCCTAAAGC | |
| Actin7 (R) | GGACAACGGAATCTCTCAGC | |
| cop1-6-S(F) GTCAACTGTCTCAATGGCTAGAAA | | |
| cop1-6-S(R) | CTATAGCCTTCCCTCCGTACTACA | |
| **Real-time qPCR** |  | |
| CSU2-real(F) | AAACAAAGCCGCAATTTCAG |  |
| CSU2-real(R) | CCTCTTTCTCTCCTTCAGTTTC |  |
| PP2A(F) | TATCGGATGACGATTCTTCGTGCAG | |
| PP2A(R) | GCTTGGTCGACTATCGGAATGAGAG | |
| **Plasmid Constructs** | |  |
| CSU2 attB1 | GGGGACAAGTTTGTACAAAAAAAGGCTTCATGCCGCCGAAGAGAAATTTC | *pDONR221-CSU2* |
| CSU2 attB2 | GGGGACCACTTTGTACAAGAAAGCTGGGTCTCTTCGCATTACACGGTTAC |
| CSU2-coil attB1 | GGGGACAAGTTTGTACAAAAAAGCAGGCTTCGATACTTTTGCTCAAGAGACTG | *pDONR221-CSU2 coil* |
| CSU2-coil attB2 | GGGGACCACTTTGTACAAGAAAGCTGGGTCCAAAAGCTTCTTGGCAGCTTC |
| CSU2-NcoI(F) | CATGCCATGGAGATGCCGCCGAAGAGAAATTT | *pSY728-YFPn-CSU2* |
| CSU2-NotI(R) | AAGGAAAAAAGCGGCCGCGTTCTTCGCATTACACGGTTAC |
| COP1-NcoI(F) | CATGCCATGGAGGAAGAGATTTCGACGGATCCGG | *pSY738-YFPc-COP1* |
| COP1-NotI(R) | AAGGAAAAAAGCGGCCGCGTCGCAGCGAGTACCAGAACTTTG |
| CSU2- EcoRI(F) | CCGGAATTCATGCCGCCGAAGAGAAATTTCAG | *pLexA-CSU2* |
| CSU2-XhoI(R) | CCGCTCGAGTTATCTTCGCATTACACGGTTACG |
| CSU2-coil-EcoRI(F) | CCGGAATTCGATACTTTTGCTCAAGAGACTG | *pLexA-CSU2 coil* |
| CSU2-coil-XhoI(R) | CCGCTCGAGCAAAAGCTTCTTGGCAGCTTC |
| ProCSU2-KpnI (F) | CGGGGTACCTTCGCCAAGCAAATGACTTGTC | *pCombia1301-proCSU2-GUS* |
| ProCSU2-NcoI (R) | CATGCCATGGCGCAGCCAGAAACTATCGGAT |
| COP1-SpeI(F) | GGACTAGTATGGAAGAGATTTCGACGGATCCG | *pCombia1300-COP1-Flag* |
| COP1-HindIII(R) | CCCAAGCTTCGCAGCGAGTACCAGAACTTTG |
